# Large-scale array of squeezed light and synchronization using atomic vapor

**DOI:** 10.1038/s41377-026-02428-w

**Published:** 2026-08-26

**Authors:** Lin Wang, Xichang Zhang, Konstantin Manannikov, Nir Davidson, Ying Hu, Dongdong Hao, Yanhong Xiao

**Affiliations:** 1 State Key Laboratory of Surface Physics, Key Laboratory of Micro- and Nano-Photonic Structures (Ministry of Education) and Department of Physics, Fudan University, Shanghai, China; 2 National Key Laboratory of Infrared Detection Technologies, Shanghai, China; 3 Shanghai Institute of Technical Physics, Chinese Academy of Sciences, Shanghai, China; 4 Department of Physics of Complex Systems, Weizmann Institute of Science, Rehovot, Israel; 5 State Key Laboratory of Quantum Optics Technologies and Devices, Institute of Laser Spectroscopy, Shanxi University, Taiyuan, China; 6 Collaborative Innovation Center of Extreme Optics, Shanxi University, Taiyuan, China

**Keywords:** Quantum optics, Atom optics

## Abstract

Quantum light sources such as squeezed light are essential for quantum information science and technologies, but the scalable production of multiple beams of them remains a challenge. Here, we experimentally demonstrate a novel approach to the generation of a large spatial array of polarization-squeezed light beams via atomic-coherence-enhanced nonlinear optical processes using a single atomic vapor cell. Unlike schemes based on independent squeezing generators, the squeezing dynamics of each channel here are governed by a common collective ground-state atomic coherence, produced by all input beams, homogenized by the thermal motion of the atoms, and protected against wall collisions by a paraffin coating. Consequently, the optical states of all channels are coupled and regulated by each other via the moving atoms, leading to synchronization behavior. We realized a 30-beam array of polarization squeezed state with 2.03 ± 0.02 dB of squeezing, experimentally verified the synchronization, and observed improved purity of the squeezed state as well as the system’s response to perturbations when the size of the array increases. This work provides a pathway towards scalable high-performance quantum light sources for applications in precision measurement, quantum imaging and quantum information processing.

**Figure:**
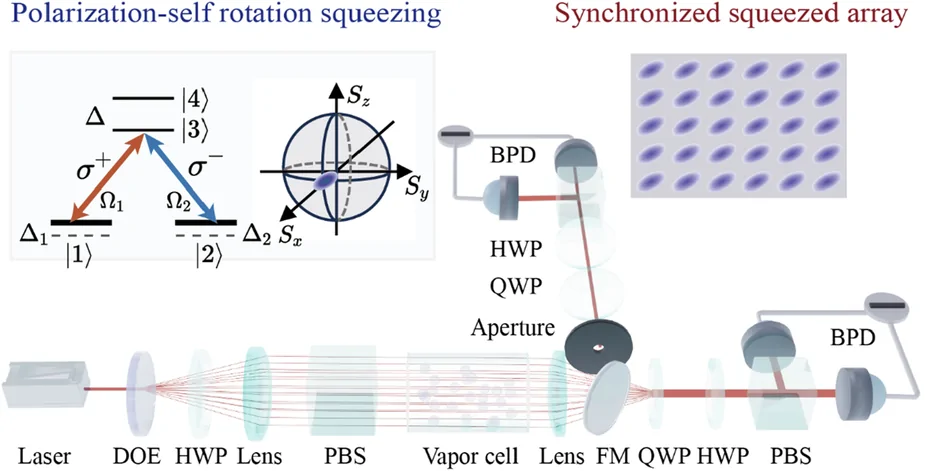

## Abstract (web summary)

A 30-beam array of polarization-squeezed light with 2.03 dB squeezing and synchronized behavior is observed via collective atomic coherence in a single vapor cell, enabling scalable quantum light sources.

## Introduction

Squeezed light is a fundamental resource for quantum networks^1,2^, imaging and sensing^3,4^, and computing^5–11^, and there is currently a demand for scalable production of many beams of it. Nowadays, multi-mode squeezed light is mainly generated using optical parametric oscillators (OPOs) with crystals in cavities^12,13^, with time- or frequency-multiplexed architectures^2,14,15^, or multiple setups^16^. Integrated photonic platforms provide an alternative route toward scalable non-classical light generation^17–19^, offering compactness and chip-level integration, though predominantly operate near telecommunication wavelengths, and scaling the number of spatial channels remains constrained. Atomic systems constitute a complementary platform for squeezed-light generation^20^, with the advantage of frequency matching with downstream atomic devices such as quantum memory and quantum sensors^21–23^. Multiple quantum optical fields^24–26^ from a single atomic vapor cell have been produced using coherence transport^24,27,28^ or multiwave mixing^29^. Recent work has further shown that vacuum vapor cells can yield high squeezing levels in a single beam^30,31^. Nevertheless, a large-scale spatial array of squeezed light remains unexplored. Beyond the potential for scaling, a shared ground-state atomic coherence across spatial channels introduces an intrinsic coupling among the modes, providing foundation for emergent collective behaviors, such as synchronization.

Synchronization is an intriguing and ubiquitous phenomenon in both natural and artificial systems^32^, where coupled oscillators develop aligned dynamical responses through mutual interactions, playing crucial roles in applications such as communication and signal processing. Among the various coupling mechanisms, dissipative coupling, in which oscillators interact dissipatively through a shared environment rather than through direct coherent exchange, provides a particularly robust route to collective behavior. Owing to its close connection with collective dynamics and correlations, synchronization in quantum systems has recently attracted significant attention and has been explored in a range of platforms, including optomechanical oscillators, trapped ions and quantum oscillators^33–38^. As a textbook model for oscillators, optical fields such as laser arrays have been found to synchronize in phase through either dissipative or coherent coupling^39–41^. Extending such collective dynamics to quantum optical fields would open a route to robust multi-channel non-classical light generation, with potential for scalable quantum technologies.

In this article, we demonstrate a large-scale spatial array of dissipatively coupled optical channels of squeezed light fields, driven by common collective atomic spin coherence in a vapor cell which can also display synchronization behavior. An array of thirty linearly polarized classical laser beams are all turned into polarization squeezed states with up to 2.03 ± 0.02 dB of squeezing, when the orthogonal polarization changes from a coherent vacuum state to a squeezed vacuum upon interacting with the atoms via a coherence-enhanced low-power nonlinear optical process. Due to the thermal atomic motion and the coherence-preserving wall coating of the glass cell, the shared coherence is jointly created by the laser array within the entire cell volume and hence enables the scalability and synchronization of the squeezed light array. We experimentally verified the uniformity in the degree of squeezing, squeezing angle (determined by the phase of the squeezed vacuum) across the array, as well as the synchronization of beams with different laser powers. Furthermore, as the size of the array increases, a higher level of squeezing (before saturation), reduced sensitivity to a defect beam, and enhanced purity of the squeezed state are observed.

## Results

### Theory of squeezed light array

**Fig. 1:**
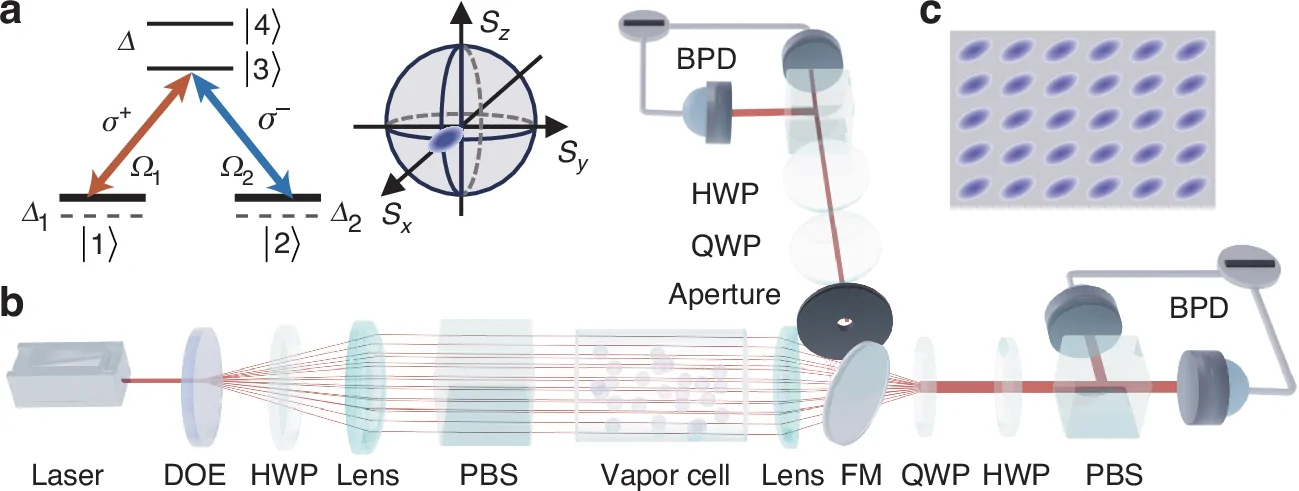
Working principles and experimental schematics of a squeezed light array. **a** Left: Simplified atomic energy levels for squeezing. The linearly polarized light drives the ^87^Rb D_1_ transition, resonantly from $\left\vert 1\right\rangle$, $\left\vert 2\right\rangle$ (belonging to 5*S*_1/2_, *F* = 2 Zeeman manifolds) to $\left\vert 3\right\rangle$ ($5{P}_{1/2},{F}^{{\prime} }=1$), and off-resonantly to $\left\vert 4\right\rangle$ ($5{P}_{1/2},{F}^{{\prime} }=2$). Here, *Ω*_1,2_ are the Rabi frequencies for the two circularly polarized component respectively, Δ = 814.5 MHz is the frequency spacing between the two excited states. Δ_1,2_ are the AC-stark shifts due to the upper excited state. Right: A Poincaré sphere illustrating the polarization squeezed state. **b** Experiment setup. A diffractive optical element (DOE) generates 30 spatially separated optical beams propagating in parallel through a coated vapor cell containing ^87^Rb atoms. The squeezed light array is characterized via two detection schemes: (i) full array quantum fluctuation measurement using balanced homodyne detection, or (ii) single-channel noise power measurement by selecting individual beams with a flip mirror (FM) while blocking others with an aperture. HWP: half-wave plate, PRP: phase-retarding plate, a quarter-wave plate (QWP), PBS: polarization beam splitter, BPD: balanced photo-detectors. **c** An illustration of synchronized squeezed ellipse of the array, where all beams have the same squeezing level and squeezing direction owing to global coupling

Our light squeezing is based on an established technique called polarization self-rotation (PSR)^30,42–44^, which can be described by a simplified four-level atom interacting with a linearly polarized (along *x*) laser beam. As shown in Fig. 1a, a *Λ*-type electromagnetically induced transparency (EIT)^45^ is formed between an excited state $\left\vert 3\right\rangle$ and two ground state degenerate Zeeman levels $\left\vert 1\right\rangle$ and $\left\vert 2\right\rangle$. With a frequency difference Δ to $\left\vert 3\right\rangle$, the upper excited state $\left\vert 4\right\rangle$ induces AC-stark shift ${\Delta }_{1,2}={\Omega }_{1,2}^{2}/\Delta$ to $\left\vert 1\right\rangle$ and $\left\vert 2\right\rangle$ respectively, where *Ω*_1,2_ is the Rabi frequency of the *σ*^+,−^ components of the input laser. This causes a polarization rotation denoted by the light’s Stokes vector component (see Supplementary Information) proportional to ${S}_{z}=({\Omega }_{1}^{2}-{\Omega }_{2}^{2})/({\Omega }_{1}^{2}+{\Omega }_{2}^{2})$, which describes a one-axis-twisting (OAT) dynamic ${S}_{z}^{2}$ for the light spin *S* on the Poincaré sphere^44,46^, and leads to shearing and squeezing of the uncertainty ellipse that determines both the squeezing level and the squeezing angle, or vacuum squeezing in the orthogonal *y*-polarization. We emphasize that the polarization rotation is enhanced by the ground state atomic coherence (due to EIT) which depends on the laser power. This process can be also understood as a degenerate four-wave-mixing (FWM) process, where the *x*-polarized input (pump) field couples the two orthogonal superposition ground states $\left\vert 1\right\rangle +\left\vert 2\right\rangle$, $\left\vert 1\right\rangle -\left\vert 2\right\rangle$ to $\left\vert 3\right\rangle$ and $\left\vert 4\right\rangle$ respectively, and produce a pair of quantum-correlated *y*-polarized photons. This type of squeezed light has been studied extensively in vapor cell systems, mostly with a single squeezed light beam^47,48^ and also in the context of quantum-enhanced atomic magnetometers^9,46,49^.

In the current system of a large laser array interacting with warm atoms in a coated vapor cell, squeezing of each optical channel is enhanced or even dominated by the common ground-state atomic coherence. This coherence is produced collectively by all laser beams and homogenized by the fast motion of the atoms, where the key is that the paraffin coating on the inner wall of the vapor cell^50^ protects the coherence against thousands of atom-wall collisions. The collective spin state lowers the laser power threshold per optical channel for squeezing and synchronizes the phases and the squeezing levels of all the squeezed vacuum. To describe the array dynamics, we treat each illuminated channel as a local double-*Λ* atom-light interaction region, while the dark region un-illuminated by light acts as a shared and dynamically-evolving reservoir of long-lived atomic coherence. Atoms acquire ground-state coherence in one optical channel, then leave the beam, travel and evolve in the dark region, and later randomly re-enter the array. The inter-channel coupling is therefore global and dissipative. In the linear polarization basis of the laser propogating along *z*-axis, the atom-light interaction in the *i*-th channel is

1 $$\begin{array}{l}{\hat{H}}_{int}=\frac{\hslash {N}_{i}}{L}\int_{0}^{L}dz\left[{\delta }^{{\prime} }{\hat{\rho }}_{33}+({\delta }^{{\prime} }+\Delta ){\hat{\rho }}_{44}\right.\\ \quad\quad\quad\,\,\,-{g}_{2}\alpha {\hat{\rho }}_{3y}-{g}_{1}\alpha {\hat{\rho }}_{4x}-{g}_{2}{\hat{a}}_{y}{\hat{\rho }}_{3x}\\ \quad\quad\quad\,\,\,\left.-{g}_{1}{\hat{a}}_{y}{\hat{\rho }}_{4y}+{{\rm{H}}}.{{\rm{c}}}.\right]\end{array}$$

where *ℏ* is the reduced Planck constant, *N*_*i*_ is the atom number of *i*-th channel, *L* is the length of the atomic medium, *g*_1,2_ are the single-photon coupling strengths, *α* is the amplitude of the *x*-polarized drive, ${\delta }^{{\prime} }$ is the one-photon detuning, and Δ is the excited-state hyperfine splitting. The coupled atomic dynamics are described by

2 $$\begin{array}{l}{\dot{\hat{\rho }}}^{(i)}=-\frac{i}{\hslash }[{\hat{H}}_{int}^{(i)},{\hat{\rho }}^{(i)}]+{\hat{{{\mathscr{L}}}}}^{(i)}[{\hat{\rho }}^{(i)}]\\ -{k}_{i0}{\hat{\rho }}^{(i)}+{k}_{i0}{\hat{\rho }}^{(0)}+{\hat{{\mathfrak{F}}}}_{i}\end{array}$$

3 $$\begin{array}{l}{\dot{\hat{\rho }}}^{(0)}=-{\gamma }_{0}{\hat{\rho }}^{(0)}+{\hat{{{\mathscr{L}}}}}^{(0)}[{\hat{\rho }}^{(0)}]\\ \qquad\quad\,\, +{\sum }_{i=1}^{N}{k}_{0i}{\hat{\rho }}^{(0)}-{\sum }_{i=1}^{N}{k}_{0i}{\hat{\rho }}^{(i)}+{\hat{{\mathfrak{F}}}}_{0}\end{array}$$

where ${\hat{\rho }}^{(i)}$ and ${\hat{\rho }}^{(0)}$ denote the atomic density matrices in the optical channels and in the dark region, respectively, ${\hat{{{\mathscr{L}}}}}^{(i)}$ includes relaxation and repopulation processes, ${k}_{i0}=\frac{d{N}_{i}}{{N}_{i}dt}$ and ${k}_{0i}=\frac{d{N}_{i}}{{N}_{d}dt}$ are the exchange rates between channel *i* and the reservoir, where *N*_*d*_ is the atom number in the dark region. *γ*_0_ is the decay rate of the ground state population, and ${\hat{{\mathfrak{F}}}}_{i,0}$ are Langevin noise operators. The generated *y*-polarized quantum field obeys

4 $$\begin{array}{l}\left(\frac{\partial }{\partial t}+c\,\frac{\partial }{\partial z}\right){\hat{a}}_{y}^{(i)}(z,t)=i{g}_{1}{N}_{i}\,{\hat{\rho }}_{y4}^{(i)}\\ +i{g}_{2}{N}_{i}\,{\hat{\rho }}_{x3}^{(i)}\end{array}$$

This framework shows that the effective inter-channel coupling is set by the channel-reservoir exchange rates and the atomic coherence lifetime in the dark region, which depends on the atomic density and the geometry of the laser beam and vapor cell. The channel spacing plays negligible roles in this global coupling. Our numerical model (see Supplementary Information) incorporates spontaneous emission, Doppler broadening, coherence decay and population exchange with the lower hyperfine energy level, and reproduces the experimental observations qualitatively. We next present the main experimental results obtained from the setup shown schematically in Fig. 1b, with more experimental details provided in the Materials and methods.

### Performance of a multiplexed squeezed light array

**Fig. 2:**
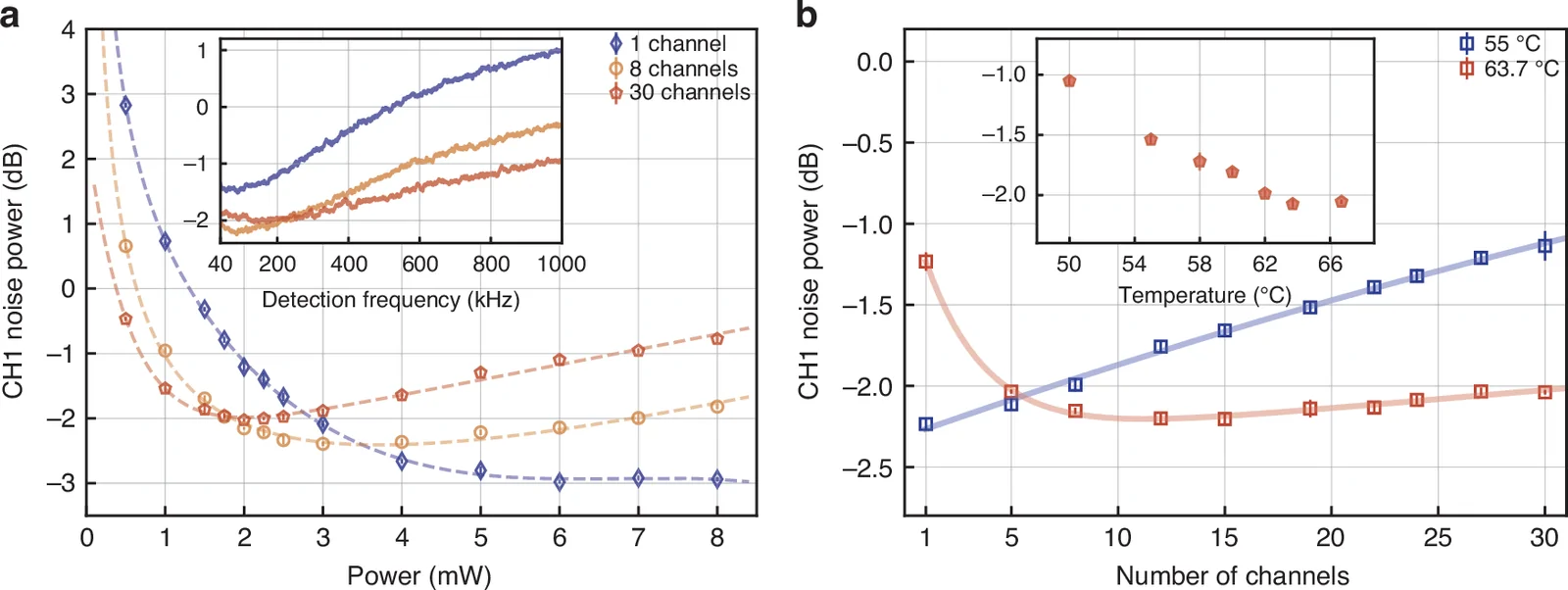
Experimental results of squeezed array. **a** Noise power of the minimal-noise-quadrature (denoted as squeezed quadrature) of a selected channel (CH1) relative to the photon shot noise level (set as 0 dB) versus the laser power per channel, for a single channel case, a 8-channel and 30-channel array respectively, while the beam size of all channel is kept at 0.505 mm in all cases. The cell temperature is 63. 7 °C. The dashed curve is a fit to guide the eye. **b** Squeezed quadrature noise of CH1 versus the array size at two temperatures (55 °C and 63. 7 °C), with a fixed input laser power of 2 mW and a corresponding beam size of 0.505 mm for each channel. The inset shows the best squeezing achievable at different cell temperatures for a 30-channel array. The corresponding optimal power per channel for maximal squeezing is [0.75 mW, 1 mW, 1.25 mW, 1.5 mW, 1.75 mW, 2 mW, 2 mW] for the temperatures of [50°C, 55°C, 58 °C, 61 °C, 62 °C, 63. 7 °C, 66. 7 °C] respectively. The solid curve is a fit to guide the eye. The shot-noise-limited performance of our detection system and laser source was verified by measuring the linear dependence of the noise power on the optical power. The shot noise level exceeded the electronic noise by approximately 20 dB within our measurement bandwidth. All noise powers are obtained at a detection frequency of 180 kHz. Spectrum analyzer settings are: resolution bandwidth (RBW) = 10 kHz, with each noise spectrum averaged for at least 40 times. Each data point represents the mean of six or more repeated measurements, and the error bars denote the standard deviation of these values

The simultaneous generation of squeezed light in a multiplexed array of 30 beams constitutes a key result of our work. We proceed to its detailed characterization by presenting the data from an arbitrarily selected channel (named CH1) as representative, with consistent performance observed across all channels. As depicted in Fig. 2a, the measured noise power for the squeezed quadrature in the 30-channel array is 2.03 dB below the photon shot noise level (set as the 0 dB reference) for a laser power of 2 mW per channel. We emphasize that, for laser power (per channel) of about 1 mW, there is no squeezing for the single-channel case, but about 1.5 dB for the 30-beam array. Even at a power as low as 0.5 mW per channel, the array generates squeezing of about 0.47 dB, whereas the single-channel case shows excessive noise of about 3 dB above the shot noise even for the minimal-noise quadrature. These results show that one can achieve squeezing in the entire array with a relatively low laser power per channel which for one channel by itself is insufficient to generate squeezing. In this regime, the system’s performance is dominated by the collective dynamics of the entire array, rather than by independent processes in individual channels. Therefore, to produce multiple squeezed beams, using a spatial array in one coated cell is superior to employing multiple vapor cells each with a single-beam, because of the better scalability and lower overall laser power.

For equal power per channel, larger arrays sustain squeezing to higher frequencies. As shown in the inset of Fig. 2a, for 2 mW incident power per channel, a representative channel (named CH1) in the 30-channel array still exhibits about 1 dB of squeezing at ~ 900 kHz and an effective bandwidth of ~ 890 kHz, compared with ~ 283 kHz for the single-channel configuration. This behavior arises from the larger total optical power in the cell, which enhances the collectively mediated atomic coherence that drives four-wave mixing. This should, however, be distinguished from the fixed-total-power comparison between arrays with different channel numbers, where distributing the same total power over more channels reduces the local pump intensity and hence both the squeezing level and bandwidth become smaller in an individual channel.

The observed achievable squeezing, however, saturates and eventually degrades for higher laser power, revealing the performance of such quantum arrays is ultimately constrained by the finite optical depth. As in any nonlinear optical process, higher power is often needed for larger nonlinearity. Here, with the assistance of atomic coherence, relatively lower laser power is required but saturation is unavoidable. As shown in Fig. 2a the squeezing level initially increases with laser power, and then plateaus and worsens. Moreover, compared to smaller arrays, the 30-channel larger array saturates at a lower power (per channel) and the achievable maximal squeezing is less. For instance, the 1-channel configuration can attain about 3 dB of squeezing (at 6 mW), while the 8-channel and the 30-channel arrays only reach 2.39 dB (at 3 mW) and 2.03 dB (at 2 mW) respectively. While increasing the total laser power in the array enhances the atomic coherence within the cell, it also introduces competing effects that limit the achievable squeezing. In the near-resonant regime, strong optical pumping redistributes population among the hyperfine ground states, transferring atoms into the *F* = 1 that do not participate in the four-wave mixing process. This population leakage lowers the effective optical depth and the four-wave mixing gain available for squeezing generation as the array size increases, leading to the observed saturation in larger arrays. At the same time, stronger resonant interaction increases spontaneous-emission-induced noise, imposing an additional limit on the achievable squeezing.

Consistent with this picture, increasing the atomic density partially compensates the reduced effective optical depth. As shown in Fig. 2b, for lower atomic density, for example at *T* = 55 °C, at a fixed 2 mW laser power per channel, squeezing is monotonically worse with increased channel number, while for *T* = 63. 7 °C, squeezing only becomes slightly worse for more than 15 channels. Furthermore, the inset shows the overall maximal squeezing level for a 30-channel array increases with vapor cell temperatures. However, further heating in our system is prohibited due to degradation of the paraffin coating, and the expected improvement at higher vapor temperatures under thermally more stable wall-coatings are theoretically analyzed in Supplementary Information. These results highlight a trade-off between mode number and per-mode squeezing in the present architecture, which prioritizes hardware-efficient generation of many spatially distinct squeezed channels from a single atomic cell, complementing cavity-based OPO and integrated photonic approaches.

### Synchronization of the squeezed light array

**Fig. 3:**
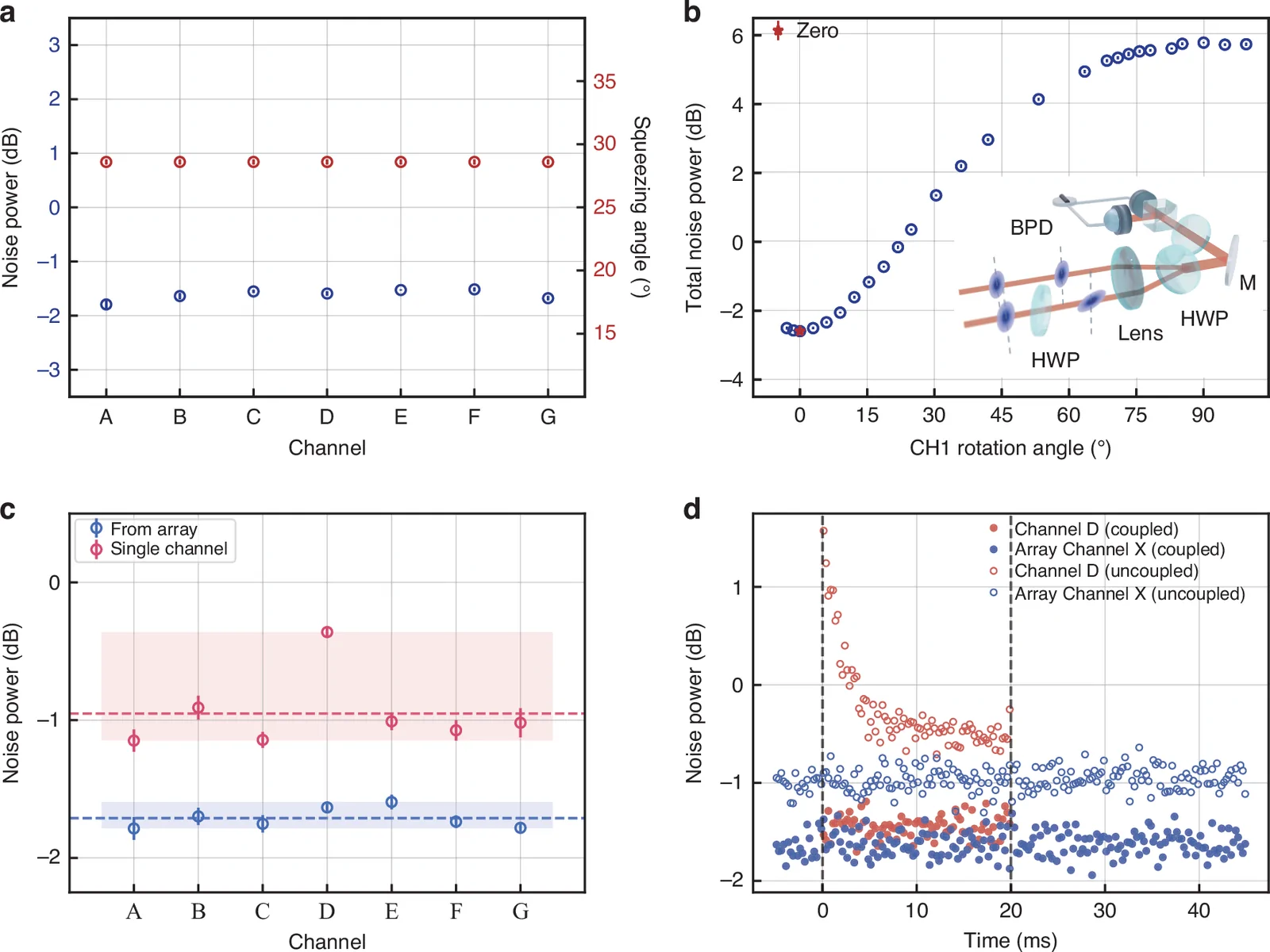
Synchronization in the squeezed light array. **a** The squeezed quadrature noise (blue) and squeezing angle (red) of seven randomly selected channels in a 30-channel array. Laser power 2 mW and beam size 0.505 mm for each channel. **b** The inset in **b** illustrates the experimental schematic at the output of the cell for measuring the total noise power using a two-channel array. One of the two squeezed beams passes through an additional quarter-wave plate (QWP), which rotates its polarization ellipse before combining with the other beam. The main panel shows the total noise power, measured via balanced homodyne detection, as a function of the rotation angle applied by the QWP. Both input squeezed beams have an optical power of 5 mW. The red pentagram denotes the noise level without rotation, while the blue points show the increase in total noise power with varying rotation angle. **c** An extra channel (Channel D) is added to the 30-channel array, where each channel of the array has a laser power of 2 mW. The laser power of Channel D is set to 1.5 mW, with a beam size identical to that of the other array channels. We measured the degree of squeezing of six channels from the array (A, B, C, E, F, G) and Channel D, under two conditions: single-channel operation case (uncoupled regime), and full 31-beam array operation case (coupled regime). The detection frequency is 186 kHz. Error bars (vertical lines in the symbol) represent standard deviations from six repetitive measurements. **d** Synchronization dynamics. Channel D, with a laser power of 1.5 mW, is operated as a pulsed beam, with the pulse starting at 0 ms and ending at 20 ms, as indicated by the black vertical dashed lines. One channel (named channel X) selected from the 30-channel continuous-wave light array (2 mW per channel) is monitored for comparison. We measured the time evolution of the degree of squeezing for both Channel D and Channel X under two conditions: single-channel operation (uncoupled regime) and full 31-beam array operation (coupled regime). The homodyne output is fed into a lock-in amplifier and demodulated at 230 kHz. The variance of the demodulated amplitude is evaluated as a function of time and used to extract the time-dependent squeezing. Channel D is pulsed at 20 Hz, and the acquisition is synchronized with the pulse trigger

Having established the squeezed array, we now explore the uniformity and synchronization in the squeezing properties, enabled by flying atoms acting as a global coupling medium inherently linking all channels within the array. We point out that, the phase of the *y*-polarized squeezed vacuum relative to that of the *x*-polarized input laser determines the orientation of the squeezed ellipse on the Poincaré sphere, or the squeezing direction. Since the phase of laser array from the DOE is the same, as experimentally proved by a far field image (see Supplementary Information), we can conclude that the squeezed vacuum array are phase synchronized if the squeezing angle is the same across the array. We provide experiment results to verify the uniform squeezing angle of the generated squeezed array. The squeezing angle, denoted by the direction *θ* of the long axis of the ellipse on the Poincaré sphere relative to the equator plane, is derived and calibrated from the phase retarder’s rotation angle (see Supplementary Information). For seven randomly selected channels, nearly identical squeezing angles and squeezing level are observed, as shown in Fig. 3a. This measurement of selecting an individual element from the array is however affected by loss due to the clipping by the aperture when selecting a certain beam (Fig. 1b).

To further prove the uniform squeezing direction, we perform additional experiments where the squeezing level of the array as a whole is detected and compared with a single channel. As shown in Fig. 3b inset, we first perform a two-channel array experiment where for one channel the phase of the *y*-polarized squeezed vacuum field is shifted via a phase retarder (QWP) before the lens, thus rotating its squeezing ellipse. The detected squeezing of the total array shown in Fig. 3b, changes with this rotation in an expected trend consistent with our theoretical simulations (see Supplementary Information): the overall squeezing is maximal when the two ellipses are aligned, with a monotonic degradation as their angular difference grows.

Then, we perform such experiments as in Fig. 3b for an array of 10 channels (laser power 1 mW per channel), but respectively let 10, 5, and 1 channels into the detector system (without mis-aligning them as above), and observe nearly identical squeezing of 1.18 dB, 1.19 dB and 1.19 dB respectively. Because of laser power limit of the photo-detectors we could not examine a larger array. These experiments prove that all the squeezed quadratures are oriented along the same direction.

Synchronization often manifests itself as unifying the initially different phases or amplitudes of many oscillators by introducing coupling between them. In our system, when channels with different laser power give different squeezing levels for the independent single-channel operation, their squeezing can be synchronized in the array operation. The key signature of the regime for synchronization here is when the squeezing level versus the laser power exhibits a plateau as shown in Fig. 2a. This plateau indicates saturation, similar to the saturation regime in classical mode-locked lasers where the system settles into a globally stable synchronized state. To verify this, we introduce an additional channel (named “D”) with 1.5 mW laser power in the 30-channel array uniform array with 2 mW per channel, and we measure squeezing of six channels (A,B,C,E,F,G) from the array and the channel D. As seen in Fig. 3c, when only one channel is present in the vapor cell, channel D shows less squeezing than others, which is consistent with the single-channel case in Fig. 2a. When all the 31 beams are in the cell, channel D has similar squeezing with the rest while the squeezing in all channels are enhanced. As indicated by the colored bar Fig. 3c, the inter-channel difference in the squeezing level is reduced from 0.79 dB to 0.19 dB when the system changes from a uncoupled to a couple regime.

We emphasize that this synchronization behavior in the saturation regime is analogous to that in both mode-locked lasers^51,52^ and phase-locked laser arrays^39^ where the synchronized mode survives because it suffers minimal loss by the saturable absorber. The synchronized state is dynamically selected out among all possible modes. In passively mode-locked lasers, the saturable absorber provides the nonlinear loss discrimination, where the high-peak-power pulse mode experiences minimal loss, thereby strongly suppressing the continuous wave mode. In coupled laser arrays^39^, the phase-locked mode corresponds to the highest total laser power focused on the saturable absorber in a cavity, also corresponding to minimal loss. In both cases, the saturation mechanism provides the critical nonlinear process required for the emergence of the synchronized mode. Here the entire atomic medium plays the role of the saturable absorber because the atoms are flying and the entire array see the same atomic ensemble, which is equivalent to focusing the whole laser array into a solid-state absorber as in ^53^. In essence, synchronization occurs when all the 31 squeezed vacuum fields are in phase and the forward scattered *y*-polarized quantum light is maximal in total (hence squeezing is maximal), which is resulted from the long-lived (minimal loss) Doppler-free collective spin state that survives.

We note that, although in principle, synchronization could also manifest through the squeezing angle, in practice due to spontaneous emission noises the squeezed state combines modest squeezing with large excessive noises especially in the anti-squeezed quadrature. This effectively smears out the uncertainty ellipse and renders the orientation of the noise ellipse insensitive to small variations induced by laser power changes (see Supplementary Information). As a result, when experimentally validating synchronization, we can only measure the squeezing levels, which are inherently connected to the squeezing angle in the OAT light squeezing scheme. Direct experimental proof of dynamic phase synchronization or locking in the squeezed vacuum array is not feasible in our experiment.

To further substantiate synchronization, we probe the temporal dynamics through a pulsed experiment as shown in Fig. 3d. We applied a localized pulsed pump of 20 ms in duration with laser power of 1.5 mW to an optical channel (named Channel D) outside of the 30-beam array where each channel has input laser power of 2 mW. We then monitored the time evolution of the squeezing level of Ch.D, and a channel (named Channel X) from the array for comparison. For the uncoupled case, i.e., when Ch.D (or Ch.X) is present alone in the Rb cell, one can observe the establishment of the steady state near the turn-on time *t* = 0 for Ch.D, which takes about 2.7 ms, and that the squeezing level of 0.5 dB is different than the 1 dB of Ch.X because their laser power is different. In contrast, for the coupled case, i.e., when Ch.D and Ch.X coexist with the rest of the array and they are all coupled, Ch.D’s squeezing rapidly reaches about the same level (1.5 dB) of the array with a nearly invisible transit near *t* = 0, which is a quick synchronization. The difference in the transit time for Ch.D in the coupled and uncoupled cases is due to the following. The dissipation rate of the collective coherence determines the time for the system to reach steady state upon perturbation, and this is determined by both the physical movement of the atoms containing many wall bounces (i.e., coherence transport) and the collective atom-light interaction, i.e., the same set of atoms interact with all the 30 or 31 optical channels which bring nonlinearity and saturation. The total laser power in the vapor cell for the 31-beam array (coupled regime) is much higher than for the 1-beam case (uncoupled regime), and hence larger array causes faster optical pumping process and therefore quicker dynamics.

### Study of the array’s response to imperfection

**Fig. 4:**
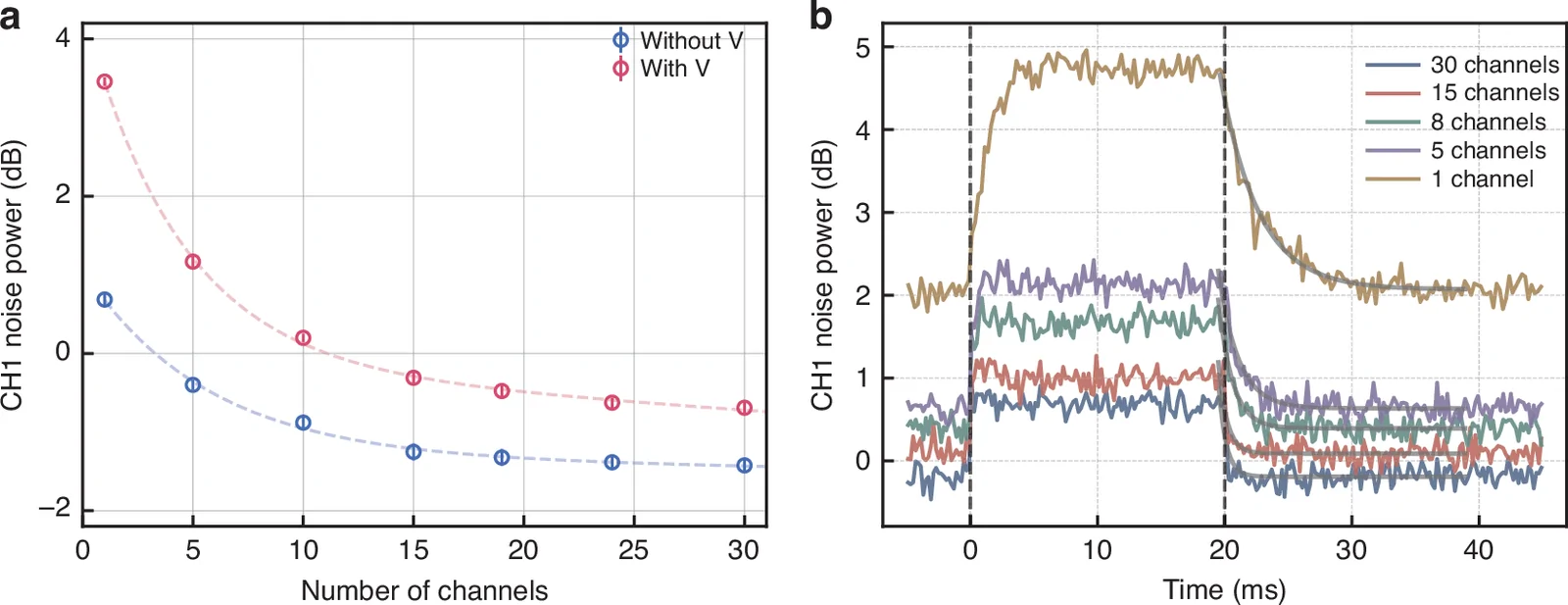
Response of the array to a defective channel. **a** Degradation of squeezing due to a “defect” channel with input light polarization (V) orthogonal to that of the array, for arrays containing different numbers of channels. The solid curve is a fit to guide the eye. The defect beam: 2 mW, waist 0.525 mm. The H-polarized beams in the array: 1 mW per channel, waist 0.505 mm. The detection frequency is 186 kHz. Error bars (vertical lines in the symbol) represent standard deviations from six repetitive measurements. **b** Time-resolved response of the array to a pulsed defect channel. The defect beam has a power of 1.0 mW and a waist of 0.468 mm, whereas the H-polarized array beams have a power of 0.5 mW per channel and a waist of 0.505 mm. The defect beam is switched on at 0 ms and switched off at 20 ms, while the H-polarized array beams remains in continuous wave operation. The squeezing level of one selected H-polarized channel in the array is recorded as a function of time using the same acquisition method as in Fig. 3d. Arrays containing 1, 5, 8, 15 and 30 channels are measured, yielding recovery times of 2.99 ± 0.09, 1.54 ± 0.08, 1.11 ± 0.05, 0.67 ± 0.06 and 0.59 ± 0.07 ms respectively (with errors from fitting uncertainties), after removal of the defect channel

Furthermore, we investigate the response of the squeezed array to imperfections, i.e., the array’s ability to mitigate the detrimental effect of a “defect” optical channel, and found that a larger array is more tolerant than a smaller one. For an array containing just two optical channels with orthogonal polarizations (otherwise with identical parameters), the coherence generated by the two channels interferes destructively^54^, thus suppressing light squeezing in both channels. Crucially, when this orthogonally polarized “defect” beam is introduced into an array, the resulting modification of the atomic spin state and destructive interference propagate through the coupled medium. This leads to a system-wide breakdown of the required ground-state atomic coherence across all channels. This global suppression of coherence is the underlying mechanism for the observed rapid degradation of squeezing throughout the entire array, strongly suggesting that the quantum noise reduction relies on a collective, highly coherent state established by all coupled channels. However, as the channel number in the array (all with horizontal ("H”) polarizations) increases, the detrimental effect of the “defect” vertically ("V”) polarized channel is reduced through motional averaging of the atoms, causing the net coherence to asymptotically approach that of a pure H-polarized array and some recovery of the squeezing. Nevertheless, the additional quantum noise introduced by the V-channel cannot be averaged out. As shown in Fig. 4a, while the degradation of H-channel squeezing due to the defect V-channel is alleviated with increasing channel number (all “H”), a residual damage remains, preventing a full recovery of squeezing.

The recovery dynamics after removal of the defect are characterized in an independent time-resolved measurement in Fig. 4b. This measurement is carried out at lower optical powers, because the dynamics become too fast to visualize at higher powers as explained above. As can be seen, the squeezing of a monitored channel from the array (named CH1) gradually recovers once the defect pulse is switched off. The recovery time decreases from 2.99 ± 0.09 ms in the single-channel array case to 0.59 ± 0.07 ms for a 30-channel array, confirming the previous analysis that larger arrays rebuild the collective coherent state faster than samller array after a perturbation.

### The purity of squeezed light state

**Fig. 5:**
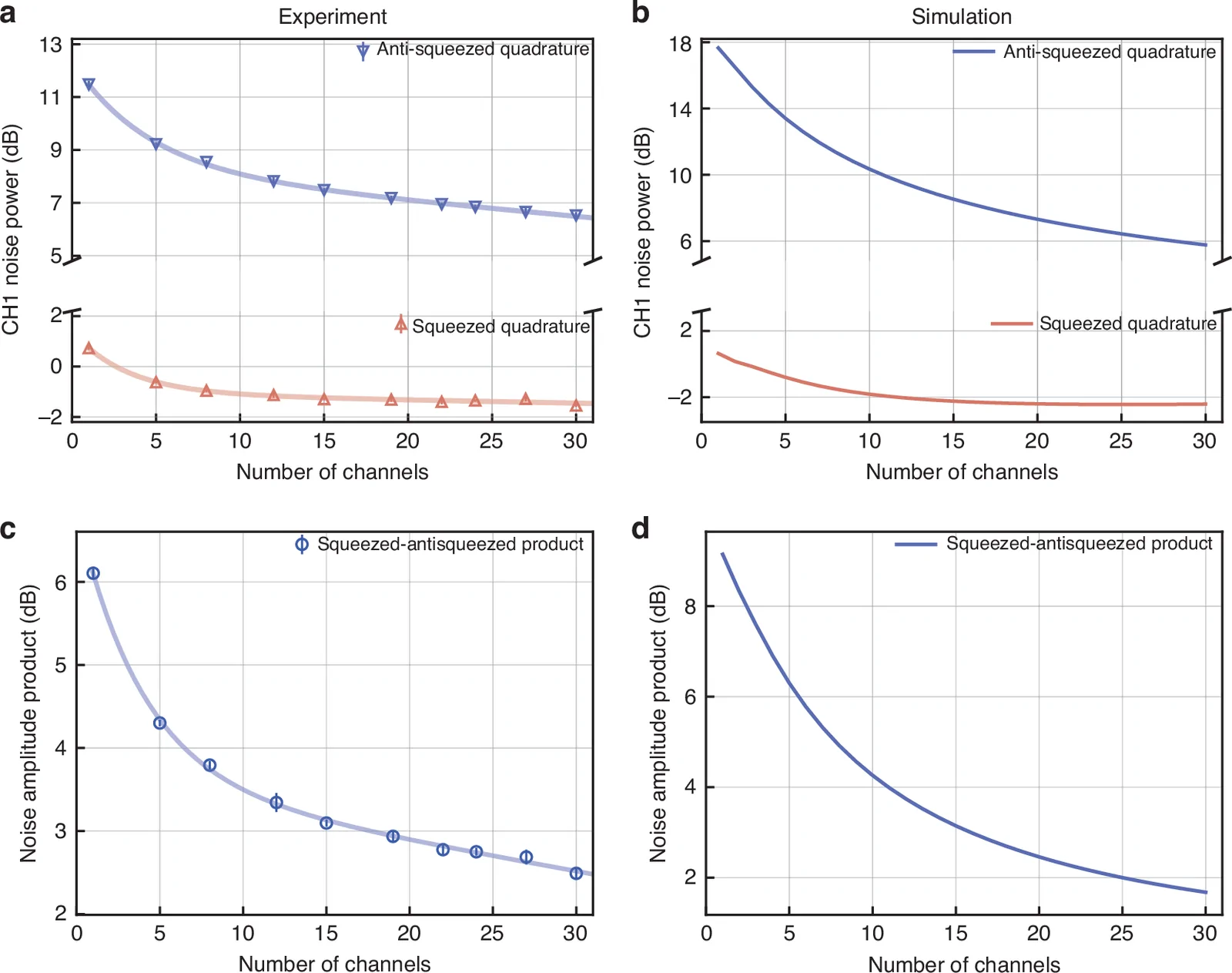
Improved purity of squeezed states for a larger array. Experimental and numerical results of the squeezed and anti-squeezed quadrature noises of a selected channel (CH1) in the array, for arrays containing different number of channels. The solid curve in **a** is a fit to guide the eye. Laser power of 1 mW and beam size of 0.505 mm for each channel. Detection frequency 180 kHz. Spectrum analyzer settings are the same as that in Fig. 2. Error bars (small vertical lines inside the symbols) represent standard deviations from six repetitive measurements. **c**, **d** show, in dB, the products of the squeezed and anti-squeezed noise amplitudes, equal to the mean of the two dB numbers in the upper **a**, **b** respectively. Zero dB corresponds to a pure unitary squeezed state. Despite the qualitative agreement between the theory and experiment, some quantitative deviation remains mainly due to neglecting experimental imperfections in the simplified model, such as inhomogeneities in the laser powers and polarizations in the array beams, optical losses, finite detection efficiency, and residual magnetic field inhomogeneity and noises etc

Finally, an interesting feature of our quantum light array is that the purity or unitarity of the squeezed light can be manipulated by leveraging the balance between squeezing and saturation. The Heisenberg uncertainty principle dictates that an ideal unitary squeezing process produces the same amount of squeezing and anti-squeezing, a property critical in harnessing quantum resources^55,56^. However, excessive anti-squeezing often appears due to dissipation processes which degrades the entanglement. Here, light scattering during squeezing^57^ induces excessive noise above the photon shot noise level. As shown in Fig. 5 this is most severe when the total laser power is low, rendering a relatively weak ground-state coherence and a negligible EIT effect. Remarkably, when the channel number increases, the initially high noise level in the anti-squeezed quadrature drastically decreases, indicating improved squeezed-state purity. This is mainly attributed to population loss to *F* = 1 states at higher total laser power in the array which reduces the absorption and associated noises. These experimental results are in good qualitative agreement with theoretical simulations (detailed in Supplementary Information) in Fig. 5b. To quantify the state purity, we also plot in Fig. 5c, d the product of the squeezed and anti-squeezed noise amplitudes, derived from the square root of the corresponding noise variances. The product decreases with increasing channel number, indicating reduced excess noise and improved purity of the squeezed states.

## Discussion

In conclusion, we have demonstrated a large-scale laser array of polarization squeezed states using a rubidium vapor cell. The squeezing level and angle of all the beams in the array are aligned due to the collectively built and shared atomic coherence by the laser array that drive the nonlinear optical process for squeezing. Synchronization behavior can be observed for moderately mismatched channels. The path to higher squeezing in our system includes using a longer cell, and a coating more resistant to higher temperatures^58,59^ with proper optimization on laser power and laser detunings, to obtain larger optical depth while maintaining the atomic coherence. Using a wider cell also helps alleviate saturation hence improves squeezing for quantitative analysis (see Supplementary Information). Another route is to incorporating a different underlying squeezing mechanism based on off-resonant atom-light interaction in presence of a magnetic field that gives light squeezing as high as about 10 dB^31^ and is compatible with our diffusively-coupled array protocol. Our approach to large-scale squeezed light array has the advantage of scalability, synchronization, tunability, tolerance to system imperfections and compatibility with downstream atomic devices.

When further improvements are implemented, this architecture could be used for spatially resolved quantum-enhanced sensing, quantum imaging, optical computing and quantum networks. In particular, although the beams in the array in our experiment are not quantum-correlated to each other^28^, through followed linear optical processing^60,61^, multipartite entanglement states can be created, and be subsequently used for quantum-enhanced distributed sensing networks^62,63^ or multi-parameter sensing^64,65^.

## Materials and methods

### Experimental setup

The experimental setup is designed to generate and characterize a multiplexed array of squeezed light beams as illustrated in Fig. 1b. A laser, tuned to the ^87^Rb ${{{\rm{D}}}}_{1}\,F=2\to {F}^{{\prime} }=1$ transition (795 nm), is split into a two-dimensional 7 × 7 array by a custom diffractive optical element (DOE) with a measured intensity uniformity of ~ 91%. Each beam has a waist (1/*e*^2^) of 0.505 mm at the cell center, with single-beam powers ranging from 0.5 to 8 mW (2 mW yields optimal squeezing for 30-channel array). The 3 mm inter-beam spacing is preserved over 0.75 m with negligible distortion. The atomic ensemble is housed in a cylindrical glass cell (2.5 cm diameter, 7.5 cm length) filled with enriched ^87^Rb. To preserve ground-state coherence, the cell inner wall is coated by paraffin that shows no degradation over years of operation below its 67 °C melting point. Experiments are conducted at an optimal temperature of 63.7 °C. The cell is placed inside a four-layer magnetic shield. Because the vapor cell is not positioned exactly at the geometric center of the four-layer magnetic shield, and the 2.5 cm-wide apertures of the shield layers are not perfectly concentric, the outer ring of the array is partially clipped, leaving only 30 fully transmitted optical channels. The noise power of the output array, with each beam consisting of the *x*-polarized pump and the generated squeezed vacuum, is measured via balanced homodyne detection (BHD) system, where the pump field itself acts as the local oscillator^66^. Two BHD systems are employed and switched via a flip mirror. To quantify the light squeezing of a beam, one BHD was used to directly measure individual channels. To probe synchronization across the array, a second BHD was configured with a lens to focus and measure a group of beams simultaneously. Details of the BHD configuration are provided in Supplementary Information.

## Supplementary information

Supplementary Information for Large-scale array of squeezed light and synchronization using atomic vapor

## Data Availability

All the data supporting the findings of this study are available within this article and its Supplementary Information. Any additional information can be obtained from corresponding authors on reasonable request.
