# Supplementary material for: Large-scale array of squeezed light and synchronization using atomic vapor
Source: Light Sci Appl. 2026 Aug 26;15:365. doi: 10.1038/s41377-026-02428-w (PMC13519026; doi:10.1038/s41377-026-02428-w)
Supplement: Supplementary file 1 [file 41377_2026_2428_MOESM1_ESM.pdf]

Supplementary Information for Large-scale array of squeezed light and synchronization using atomic vapor

Lin Wang[1], Xichang Zhang[2,3], Konstantin Manannikov[4], Nir Davidson[4], Ying Hu[5,6], Dongdong Hao[1*], Yanhong Xiao[1,5,6*]

[1]State Key Laboratory of Surface Physics, Key Laboratory of Micro- and Nano-Photonic Structures (Ministry of Education) and Department of Physics, Fudan University, Shanghai, 200433, China.
[2]National Key Laboratory of Infrared Detection Technologies, Shanghai, 200083, China.
[3]Shanghai Institute of Technical Physics, Chinese Academy of Sciences, Shanghai, 200083, China.
[4]Department of Physics of Complex Systems, Weizmann Institute of Science, Rehovot, 7610001, Israel.
[5]State Key Laboratory of Quantum Optics Technologies and Devices, Institute of Laser Spectroscopy, Shanxi University, Taiyuan, 030006, China.
[6]Collaborative Innovation Center of Extreme Optics, Shanxi University, Taiyuan, 030006, China.

*Corresponding author(s). E-mail(s): ddhao19@fudan.edu.cn; yxiao@fudan.edu.cn;

Supplementary Note 1: One-axis-twisting light squeezing by polarization self-rotation

The polarization self-rotation (PSR) effect can be described using a double-Λ four-level system, where the linearly polarized light couples the ground states ($|1\rangle$, $|2\rangle$) and the excited states ($|3\rangle$, $|4\rangle$). To simplify the model, we assume that the energy splitting Δ between $|3\rangle$ and $|4\rangle$ is large, so that when the laser is near resonant with $|3\rangle$, the

influence of $|4\rangle$ is solely AC-Stark shifts of the ground states. Consequently, in the circular polarization basis, the Hamiltonian of this system takes the simplified form:

$$H_{int} = \frac{\hbar N_0}{L}\int_0^L dz\left[\Delta_1\hat{\rho}_{11} + \Delta_2\hat{\rho}_{22} - \Omega_1\hat{\rho}_{13} - \Omega_2\hat{\rho}_{23} + \text{H.c.}\right], \tag{S1}$$

where N_0 is the number of atoms and L is the length of the atomic medium. The Rabi frequencies for the right- and left-circularly polarized pump light are $\Omega_1 = \frac{d_R E_R}{\hbar}$ and $\Omega_2 = \frac{d_L E_L}{\hbar}$ respectively, with $d_{R,L}$ being the dipole moments. The light-induced AC Stark shifts are given by $\Delta_1 = \Omega_1^2/\Delta$ and $\Delta_2 = \Omega_2^2/\Delta$. For x-polarized light, $\Omega_1 = \Omega_2 = \Omega$, and the relative phase between the right and left circular polarization components is zero, $\Omega_1^* = \Omega_1, \Omega_2^* = \Omega_2$. The system dynamics is governed by the master equation:

$$\begin{cases} \dot{\rho}_{11} = -\gamma_0\rho_{11} + i\Omega_1\rho_{13} + \gamma_0\rho_{22} - i\Omega_1\rho_{31} + \gamma\rho_{33} \\ \dot{\rho}_{12} = -\gamma_{12}\rho_{12} + i\Delta_1\rho_{12} - i\Delta_2\rho_{12} + i\Omega_2\rho_{13} - i\Omega_1\rho_{32} \\ \dot{\rho}_{13} = i\Omega_1\rho_{11} + i\Omega_2\rho_{12} - \gamma\rho_{13} + i\Delta_1\rho_{13} - i\Omega_1\rho_{33} \\ \dot{\rho}_{22} = \gamma_0\rho_{11} - \gamma_0\rho_{22} + i\Omega_2\rho_{23} - i\Omega_2\rho_{32} + \gamma\rho_{33} \\ \dot{\rho}_{23} = i\Omega_1\rho_{21} + i\Omega_2\rho_{22} - \gamma\rho_{23} + i\Delta_2\rho_{23} - i\Omega_2\rho_{33} \\ \dot{\rho}_{33} = -i\Omega_1\rho_{13} - i\Omega_2\rho_{23} + i\Omega_1\rho_{31} + i\Omega_2\rho_{32} - 2\gamma\rho_{33} \end{cases} \tag{S2}$$

where γ_0 is the decay rate of the ground state population, γ is the decay rate of optical coherence, and γ_{12} is the decay rate of ground-state coherence. The steady-state solution of the optical coherence can be derived:

$$\rho_{13} = \frac{\frac{i}{2}\Omega_1 + i\Omega_2\rho_{12}}{-i\Delta_1 + \gamma}, \quad \rho_{23} = \frac{\frac{i}{2}\Omega_2 + i\Omega_1\rho_{21}}{-i\Delta_2 + \gamma}, \tag{S3}$$

where the ground-state coherence is $\rho_{12} = \frac{-\Omega_1\Omega_2/\gamma}{\gamma_{12} + \frac{\Omega_1^2+\Omega_2^2}{\gamma} + i(\Delta_2 - \Delta_1)}$. The two optical susceptibilities are given by $\chi_1 = \frac{N_0}{\varepsilon_0 E_R}(d_{13}\rho_{13} + d_{14}\rho_{14}), \chi_2 = \frac{N_0}{\varepsilon_0 E_L}(d_{23}\rho_{23} - d_{24}\rho_{24})$, and the real part $\text{Re}(\chi)$ determines the refractive index. The phase difference between the left- and right-circularly polarized components after propagation through the atomic medium of length L is $\phi = \frac{2\pi L(n_R - n_L)}{\lambda}$, where λ is the laser wavelength. The polarization state of the laser field is characterized by the Stokes vector, and the polarization rotation can be written as:

$$\begin{aligned} S_y &\propto [\text{Re}(\chi_1) - \text{Re}(\chi_2)] \\ &\propto \frac{1}{\gamma^2 + \Delta_1^2}[(\Delta_2 - \Delta_1)(\frac{1}{2} + \text{Re}(\rho_{12})) - 2\gamma\text{Im}(\rho_{12})]. \end{aligned} \tag{S4}$$

Substituting the expression for ρ_{12} into Eq. (S4) yields:

$$\begin{aligned} S_y &\propto (\Delta_2 - \Delta_1)(1 - \frac{1}{2}\frac{\Omega^2}{(\gamma_{12} + \frac{\Omega^2}{\gamma})^2}) \\ &\propto S_z\Omega^2(1 - \frac{1}{2}\frac{\Omega^2}{(\gamma_{12} + \frac{\Omega^2}{\gamma})^2}), \end{aligned} \tag{S5}$$

where $S_z = \frac{\Omega_1^2-\Omega_2^2}{\Omega_1^2+\Omega_2^2}$. This expression provides a formal derivation of the one-axis-twisting (OAT) Hamiltonian, $H \propto S_z^2$, which generates the shearing dynamics and squeezing of the quantum noise.

Supplementary Note 2: Numerical simulation

Multi-channel numerical model

We can quantitatively evaluate the quantum noise of y-polarized photons generated through the squeezing process by the input x-linearly polarized light. The Hamiltonian for the double-Λ system in the linear polarization basis (Fig. S1(b)) can be expressed as:

$$H_{int} = \frac{\hbar N_0}{L}\int_0^L dz\big[\delta'\hat{\rho}_{33} + (\delta'+\Delta)\hat{\rho}_{44} - g_2\alpha\hat{\rho}_{3y} - g_1\alpha\hat{\rho}_{4x} - g_2\hat{a}_y\hat{\rho}_{3x} - g_1\hat{a}_y\hat{\rho}_{4y} + \text{H.c.}\big], \tag{S6}$$

where the linear polarization atomic bases are defined as $|x\rangle = (|1\rangle + |2\rangle)/\sqrt{2}$ and $|y\rangle = (|1\rangle - |2\rangle)/\sqrt{2}$. The single-photon Rabi frequency is given by $g_i = \mu_i\mathcal{E}/\hbar$, with μ_i denoting the dipole moment and $\mathcal{E}$ representing the single-photon electric field amplitude. The parameter $\alpha = E_x/\sqrt{\hbar\omega/(4\pi\epsilon_0 A)}$ characterizes the strength of the driving field, where E_x is the amplitude of the x-polarized electric field, ϵ_0 is the vacuum permittivity and A denotes the cross-sectional area of the laser beam. δ' is the one-photon detuning and Δ is the hyperfine splitting between the two upper excited states. For an N-channel array where coherence in the dark region (not illuminated by the laser array) $\rho^{(0)}$ is treated as a non-Markovian bath, the evolution equations are:

$$\begin{cases} \dot{\rho}^{(1)} &= -\frac{i}{\hbar}[H_{int}^{(1)}, \rho^{(1)}] - \frac{1}{2}\{\Gamma^{(1)}, \rho^{(1)}\} + S_1 - k_{10}\rho^{(1)} + k_{10}\rho^{(0)} + \mathfrak{F}_1 \\ \dot{\rho}^{(2)} &= -\frac{i}{\hbar}[H_{int}^{(2)}, \rho^{(2)}] - \frac{1}{2}\{\Gamma^{(2)}, \rho^{(2)}\} + S_2 - k_{20}\rho^{(2)} + k_{20}\rho^{(0)} + \mathfrak{F}_2 \\ &\vdots \\ \dot{\rho}^{(N)} &= -\frac{i}{\hbar}[H_{int}^{(N)}, \rho^{(N)}] - \frac{1}{2}\{\Gamma^{(N)}, \rho^{(N)}\} + S_N - k_{N0}\rho^{(N)} + k_{N0}\rho^{(0)} + \mathfrak{F}_N \\ \dot{\rho}^{(0)} &= -\frac{1}{2}\{\Gamma^{(0)}, \rho^{(0)}\} - \gamma_0\rho^{(0)} + S_0 - \sum_{i=1}^N k_{i0}\rho^{(0)} + \sum_{i=1}^N k_{0i}\rho^{(i)} + \mathfrak{F}_0 \end{cases} \tag{S7}$$

Here the relaxation matrix $\Gamma^{(i)}$ describes atomic decay processes, and S_i represents the corresponding re-population dynamics. The forms of S_i and $\Gamma^{(i)}$ are:

$$S_i = \begin{pmatrix} \frac{\gamma_0}{2}(\rho_{yy}^{(i)} + \rho_{55}^{(i)}) + \frac{38\gamma}{100}(\rho_{33}^{(i)} + \rho_{44}^{(i)}) & 0 & 0 & 0 & 0 \\ 0 & \frac{\gamma_0}{2}(\rho_{xx}^{(i)} + \rho_{55}^{(i)}) + \frac{38\gamma}{100}(\rho_{33}^{(i)} + \rho_{44}^{(i)}) & 0 & 0 & 0 \\ 0 & 0 & 0 & 0 & 0 \\ 0 & 0 & 0 & 0 & 0 \\ 0 & 0 & 0 & 0 & \frac{24\gamma}{100}(\rho_{33}^{(i)} + \rho_{44}^{(i)}) + \frac{\gamma_0}{2}(\rho_{xx}^{(i)} + \rho_{yy}^{(i)}) \end{pmatrix},$$

$$\Gamma^{(i)} = \begin{pmatrix} \gamma_0 & \gamma_{12} & \frac{\gamma}{2} & \frac{\gamma}{2} & \gamma_{12} \\ \gamma_{12} & \gamma_0 & \frac{\gamma}{2} & \frac{\gamma}{2} & \gamma_{12} \\ \frac{\gamma}{2} & \frac{\gamma}{2} & \gamma & \gamma & \frac{\gamma}{2} \\ \frac{\gamma}{2} & \frac{\gamma}{2} & \gamma & \gamma & \frac{\gamma}{2} \\ \gamma_{12} & \gamma_{12} & \frac{\gamma}{2} & \frac{\gamma}{2} & \gamma_0 \end{pmatrix}. \tag{S8}$$

The hopping rates between an optical channel and the dark region are given by $k_{i0} = \frac{dN_i}{N_i dt} = \frac{2\pi r_i}{\pi r_i^2} \left(\frac{m}{2\pi k_B T}\right)^{1/2} \int_0^{+\infty} e^{-\frac{m v_z^2}{2k_B T}} v_z \, dv_z \approx \frac{\bar{v}}{2r_i} \sim 10^5 s^{-1}$ and $k_{0i} = \frac{dN_i}{N_d dt} = k_{i0} \frac{r_i^2}{R_{cell}^2 - \sum_i r_i^2} \sim 10^3 s^{-1}$ respectively, where r_i is the radius of the i-th channel, R_{cell} is the radius of the vapor cell, k_B is the Boltzmann constant, v is the velocity of atoms, m is the mass of a rubidium atom, T is the temperature, N_i is the atom number in the i-th channel and N_d is the atom number in the dark region. The Langevin operator $\mathfrak{F}_{(i)}$ characterizes the environment induced fluctuations of the atomic operators, which directly drives the atomic noise dynamics and affects the quantum properties of the output light field. In addition, the evolution of the vacuum-field is governed by

$$\left(\frac{\partial}{\partial t} + c\frac{\partial}{\partial z}\right) \hat{a}_y(z,t) = \frac{1}{i\hbar} \left[\hat{a}_y(z,t), H_{int}\right]. \tag{S9}$$

For a multi-channel configuration, the propagation equations for the corresponding vacuum-field take the form:

$$\begin{cases} \left(\frac{\partial}{\partial t} + c\frac{\partial}{\partial z}\right) \hat{a}_y^{(1)}(z,t) = i g_1 N_0 \, \hat{\rho}_{y4}^{(1)} + i g_2 N_0 \, \hat{\rho}_{x3}^{(1)} \\ \left(\frac{\partial}{\partial t} + c\frac{\partial}{\partial z}\right) \hat{a}_y^{(2)}(z,t) = i g_1 N_0 \, \hat{\rho}_{y4}^{(2)} + i g_2 N_0 \, \hat{\rho}_{x3}^{(2)} \\ \qquad \vdots \\ \left(\frac{\partial}{\partial t} + c\frac{\partial}{\partial z}\right) \hat{a}_y^{(N)}(z,t) = i g_1 N_0 \, \hat{\rho}_{y4}^{(N)} + i g_2 N_0 \, \hat{\rho}_{x3}^{(N)} \end{cases} \tag{S10}$$

Once the evolution equations for both the atomic and optical field operators are established, the system dynamics can be investigated in detail. To study the quantum fluctuations of the light field, we then linearize the dynamics around the steady state by expanding the operators as $\hat{\rho}_{\mu\nu}^i = \langle \hat{\rho}_{\mu\nu}^i \rangle + \delta\hat{\rho}_{\mu\nu}^i$ and $\hat{a}_y = \langle \hat{a}_y \rangle + \delta\hat{a}_y$, where $\langle \hat{a}_y \rangle = 0$. Substituting these into the evolution equations and transforming to the Fourier domain, we solve for the fluctuation $\delta\hat{a}_y(\omega)$ in terms of the atomic operators. The numerical solution yields the spectrum of the output vacuum field, which contains the quantum noise contributions from both the atomic and field fluctuations. The squeezing is quantified by the noise spectrum of a general quadrature, $\hat{X}_\theta = \hat{X}\cos\theta + \hat{P}\sin\theta$, where $\hat{X} = (\hat{a}_y + \hat{a}_y^\dagger)/2$ and $\hat{P} = (\hat{a}_y - \hat{a}_y^\dagger)/(2i)$ are the amplitude and phase quadratures, respectively. The noise spectrum of a given quadrature $\hat{X}_\theta$ can be obtained from the Fourier transform of its auto-correlation function

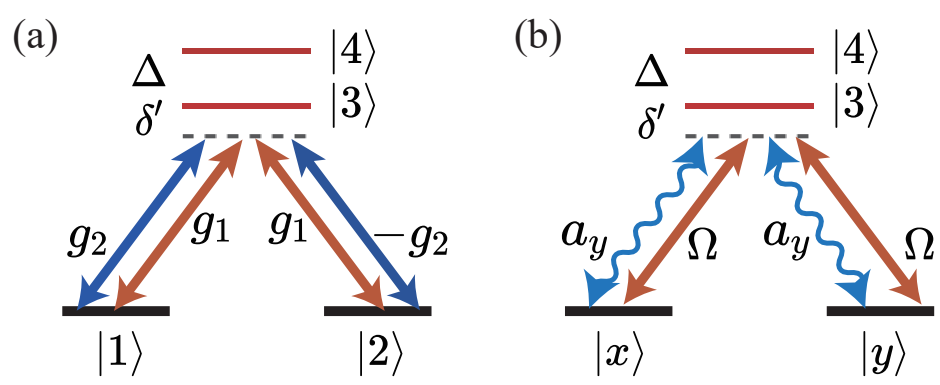

Fig. S1 The double-Λ theoretical model. (a) In the circular basis, the states $|1\rangle$ and $|2\rangle$ represent the Zeeman sublevels of the ground state $|5S_{1/2}, F=2\rangle$. g_1 is the coupling strength for the transition $|5S_{1/2}, F=2\rangle \to |5P_{1/2}, F'=1\rangle$, and g_2 for the transition between $|5S_{1/2}, F=2\rangle$ and $|5P_{1/2}, F'=2\rangle$. (b) In the linear polarization basis, Ω is the Rabi frequency of the x-polarized pump field, and a_y corresponds to the quantum field with y-polarization.

$S_{X_\theta}(\omega) = \left\langle \hat{X}_\theta(\omega)\, \hat{X}_\theta(\omega') \right\rangle$. The minimum value of $S_{X_\theta}(\omega)$ at the angle θ identifies the squeezing level at frequency ω, and the corresponding θ_{sq} defines the squeezing angle.

To accurately model the atomic dynamics and squeezing generation in the rubidium vapor cell, we extend the standard double-Λ model to include an auxiliary energy level that accounts for population loss induced by optical pumping. This is necessary since the actual ^{87}Rb atoms feature an additional hyperfine level $|F=1\rangle$ that remains uncoupled with the applied optical field. For a four-level model, the Hamiltonian is:

$$H_{int} = -\hbar \begin{pmatrix} 0 & 0 & \hat{a}_y\, g_1 & \sqrt{2}\,\alpha\, g_2 \\ 0 & 0 & \sqrt{2}\,\alpha\, g_1 & \hat{a}_y\, g_2 \\ \hat{a}_y^\dagger\, g_1 & \sqrt{2}\,\alpha\, g_1 & -\delta & 0 \\ \sqrt{2}\,\alpha\, g_2 & \hat{a}_y^\dagger\, g_2 & 0 & -(\delta+\Delta) \end{pmatrix}; \tag{S11}$$

while for a five-level model including the auxiliary state with a hyperfine splitting of $\Delta_{HF} = 6.8$ GHz, the Hamiltonian becomes:

$$H_{int} = -\hbar \begin{pmatrix} 0 & 0 & \hat{a}_y\, g_1 & \sqrt{2}\,\alpha\, g_2 & 0 \\ 0 & 0 & \sqrt{2}\,\alpha\, g_1 & \hat{a}_y\, g_2 & 0 \\ \hat{a}_y^\dagger\, g_1 & \sqrt{2}\,\alpha\, g_1 & -\delta & 0 & 0 \\ \sqrt{2}\,\alpha\, g_2 & \hat{a}_y^\dagger\, g_2 & 0 & -(\delta+\Delta) & 0 \\ 0 & 0 & 0 & 0 & 2\pi\times\Delta_{HF} \end{pmatrix}; \tag{S12}$$

In our numerical simulations, we use a Rb cylindrical vapor cell with a diameter of 2.5 cm and length of 7.5 cm, a decay rate of $\gamma_0 = \gamma_{12} = 10$ Hz, and a Doppler width of $\Delta_D = 500$ MHz, unless otherwise stated. The model describes the intrinsic squeezed-state generation from the nonlinear atom-light interaction and does not include external optical loss or technical noise. It therefore reproduces the main physical trends only qualitatively, while some deviation in the absolute values from the experimental data is expected.

Effects of Doppler broadening on squeezing angle in four-level model

Both theoretical and experimental results confirm the presence of substantial excess noise above the shot-noise limit, especially in the anti-squeezed quadrature, contrasting

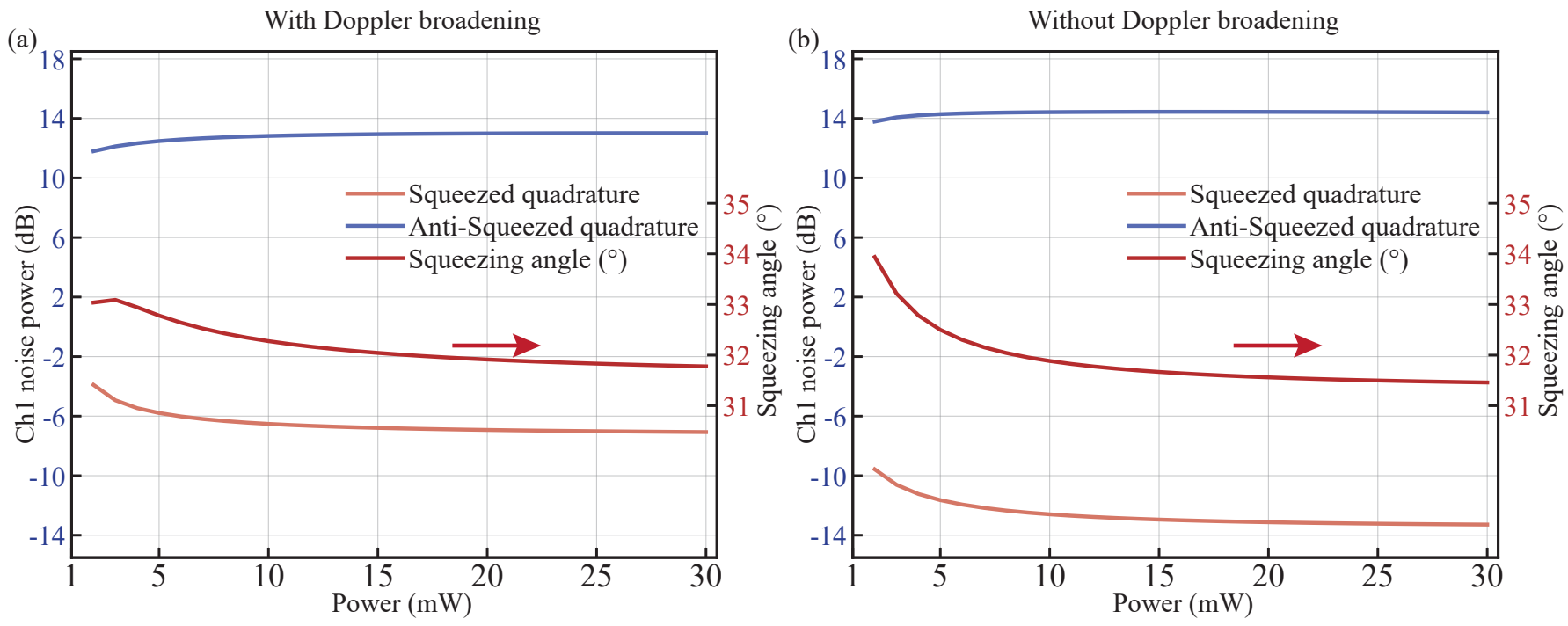

Fig. S2 Four-level model numerical results for the single-channel case, showing the squeezed and anti-squeezed quadrature noise powers and the squeezing angle as a function of laser power. (a) Four-level atomic model with Doppler broadening included. (b) Four-level atomic model without Doppler broadening. The cell temperature is set at 63.7°C, and the laser frequency is set to be 80 MHz red-detuned from $|5S_{1/2}F = 2\rangle \rightarrow |5P_{1/2}F' = 1\rangle$. Cell length: 7.5 cm; diameter: 2.5 cm. A laser power of 1 mW corresponds to a Rabi frequency of 43.6 MHz under the theoretically chosen parameters. The detection frequency is fixed at 160 kHz, and the noise power is normalized to the shot noise level (0 dB level).

with the balanced degree of squeezing and anti-squeezing a unitary squeezed state. This excess noise arises primarily from dissipative processes, particularly spontaneous emission of the atoms associated with the absorption of light. When the excess noise dominates, the small variation of the squeezing angle with experimental parameters cannot be detected. To verify this, we calculate the quantum noise and the squeezing angle with and without Doppler broadening of the excited states, within the four-level model and the single-channel case. Doppler broadening is included by integrating the system's response over the Maxwell-Boltzmann velocity distribution of the atoms. In presence of Doppler broadening, EIT efficiency is lower and the spontaneous emission introduces more excess noise. As illustrated in Fig. S2(a) and (b), one can observe that as the laser power changes, the squeezing angle varies accordingly. As expected, however, the variation is more pronounced without Doppler broadening, where the squeezed state is also closer to a unitary one, exhibiting lower excess noise in the anti-squeezed quadrature.

Comparison of four-level and five-level models

To further demonstrate that light absorption is the source of excess noise, we compare the four-level and five-level models. While the four-level model accurately captures the mechanism of squeezing generation in the atomic system, the actual atomic ground state includes both $|F = 1\rangle$ and $|F = 2\rangle$ hyperfine levels, indicating that the five-level model is more realistic. Due to the auxiliary state in the five-level model that can trap the leaked population via optical pumping, light absorption for high laser power is reduced compared to that in the four-level model under the same parameters. For comparison between the two models, the calculated laser power dependence of the quantum noise for the single channel case is presented in Fig. S3(a) and (b). In our

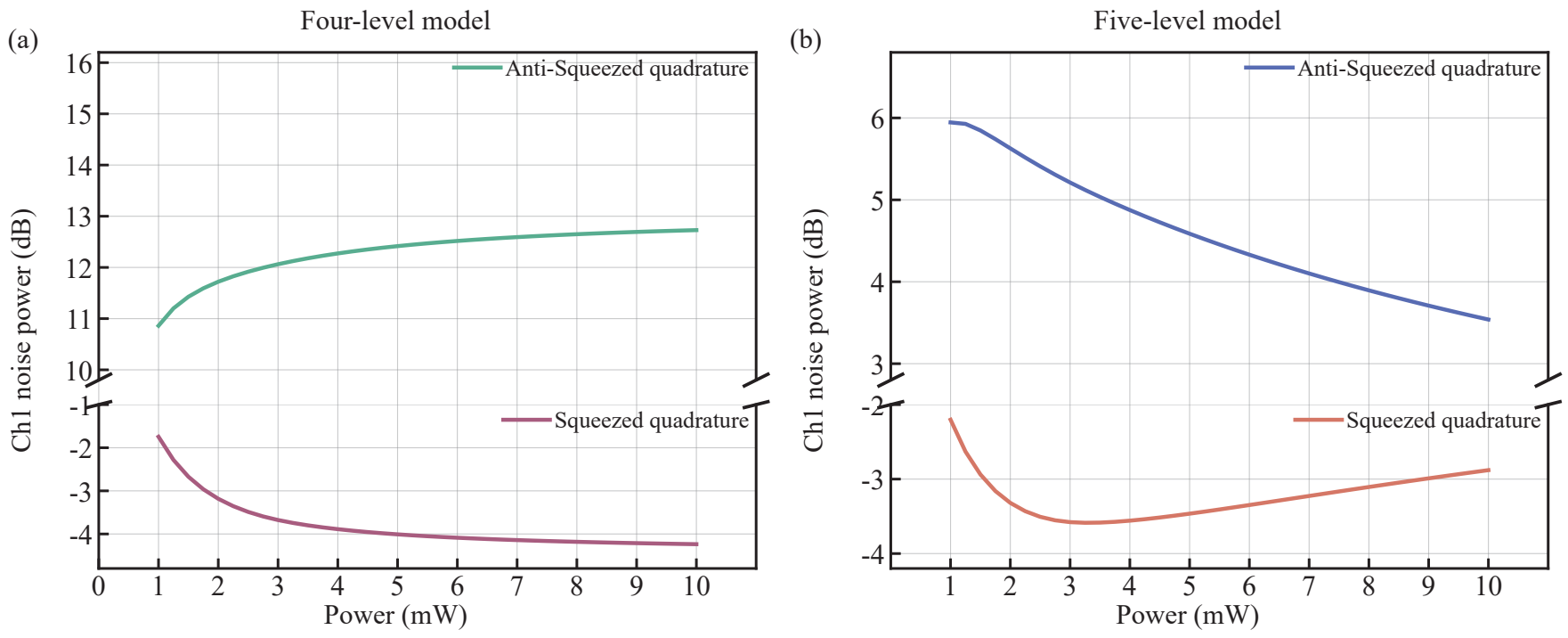

Fig. S3 Numerical results of squeezed and anti-squeezed quadratures in the single-channel case as a function of the laser power. (a) The four-level model results. (b) The five-level model results (three ground states and two excited states, while an auxiliary ground state acts as the population trapping state). The cell temperature is set at 63.7°C, and the laser is on resonant with $|5S_{1/2}F = 2\rangle \rightarrow |5P_{1/2}F' = 1\rangle$. A laser power of 1 mW corresponds to a Rabi frequency of 43.6 MHz under the theoretically chosen parameters. Cell length: 7.5 cm; diameter: 2.5 cm. The detection frequency is fixed at 160 kHz, and the noise power is normalized to the shot noise level (0 dB level).

calculations, identical parameters are employed in the two models for direct comparison. The temperature is chosen to match that used in the experiment. The decay rates of ground-state population and coherence are set to 10 Hz, where we assume that the ground-state coherence decay occurs in the dark region due to wall collisions and small magnetic field inhomogeneity. Our calculations account for Doppler broadening and use an excited-state decay rate of 6 MHz. The four-level model results (Fig. S3(a)) demonstrate the characteristic PSR enhancement with increasing power, accompanied by simultaneous increase of the degree of squeezing and anti-squeezing, where their asymmetric deviations from the photon shot noise level are due to the excess atomic noise. In contrast, the five-level model in Fig. S3(b) exhibits a distinct behavior: the anti-squeezing noise decreases with increasing optical power, in agreement with our experimental observations. The drop in the quantum noise in the anti-squeezed quadrature can be attributed to more pronounced population leakage from the $|F = 2\rangle$ state at higher laser powers, which reduces the effective atom number participating in the light-atom interactions, consequently suppressing the atomic noise contribution to the anti-squeezed component.

Performance of the squeezed array

Based on the five-level model, we perform numerical simulations for the array while keeping all other parameters identical to those described above for the single-channel case in Fig. S3. Fig. S4 shows the squeezing versus laser power for single-channel, 8-channel, and 30-channel configurations, which agree well with the experimental results in Fig. 2(a) of the main text. For equal incident laser power per channel, the required power for a specific level of squeezing decreases with increasing array size, while larger arrays exhibit earlier saturation due to higher total laser power.

The numerical results shown in Fig. S4 reveal that the maximum achievable squeezing in large-scale arrays is lower, compared to the single-channel configuration. This

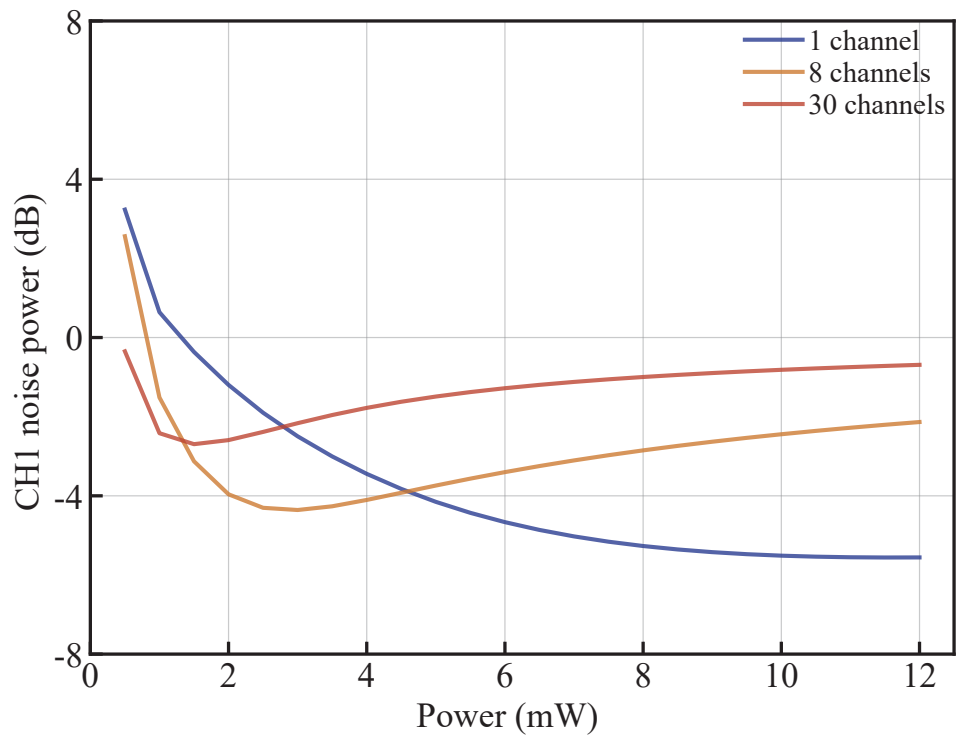

Fig. S4 Numerical results of the squeezed quadrature noise power of CH1 as a function of laser power in one-, eight-, and thirty-channel arrays, where each channel in the array has identical input laser power. The temperature is 63.7°C, with the laser frequency set to be on resonance with $|5S_{1/2}F = 2\rangle \rightarrow |5P_{1/2}F' = 1\rangle$. Cell length: 7.5 cm; diameter: 2.5 cm. A laser power of 1 mW corresponds to a Rabi frequency of 44.2 MHz under the theoretically chosen parameters. The detection frequency is fixed at 160 kHz, and the noise power is normalized to the shot noise level (0 dB level).

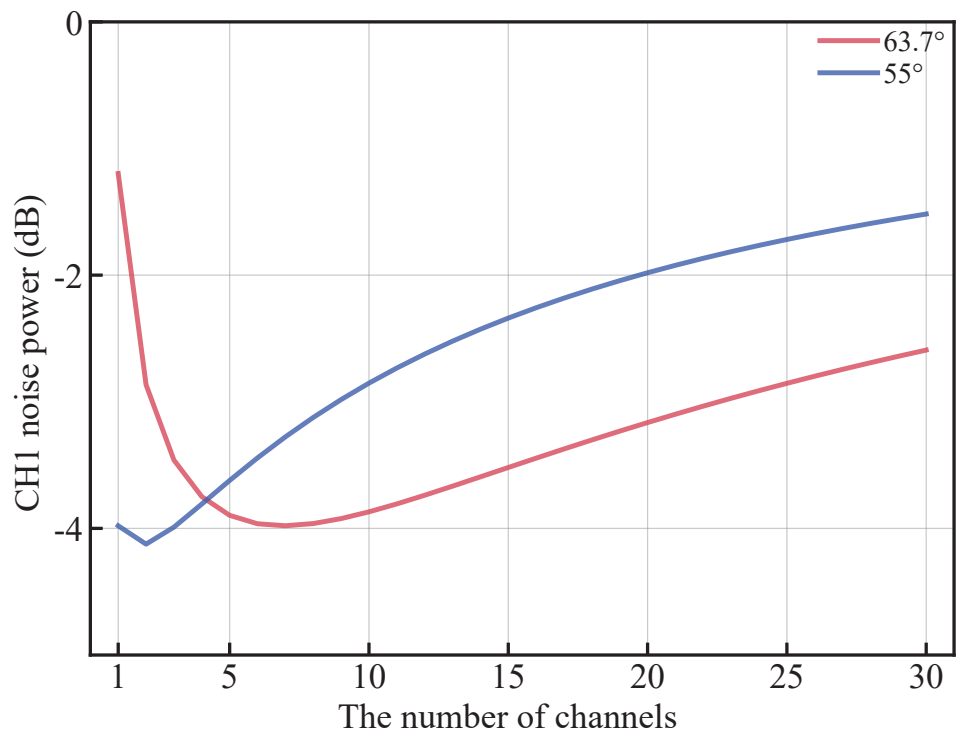

Fig. S5 Numerical results of the squeezed quadrature noise power of CH1 as a function of the array size at temperatures of 63.7°C and 55°C, where each channel in the array has the same input power of 2 mW. The laser frequency is set to be on resonant with $|5S_{1/2}F = 2\rangle \rightarrow |5P_{1/2}F' = 1\rangle$. A laser power of 2 mW corresponds to a Rabi frequency of 62.6 MHz under the theoretically chosen parameters. Cell length: 7.5 cm; diameter: 2.5 cm. The detection frequency is fixed at 160 kHz, and the noise power is normalized to the shot noise level (0 dB level).

is limited by the total number of atoms available, as experimentally verified by the results presented in the inset of Fig. 2(b) in the main text. As shown in Fig. S5, our theoretical results also illustrate the dependence of quantum noise on the array size at 63.7°C and 55°C, in agreement with the experimental results in the main text. At higher temperatures, saturation with array size is delayed as a result of the increase in total atom number, and the squeezing performance for a large array improves with temperature. This suggests that increasing temperature is a practical route for optimizing squeezing in array.

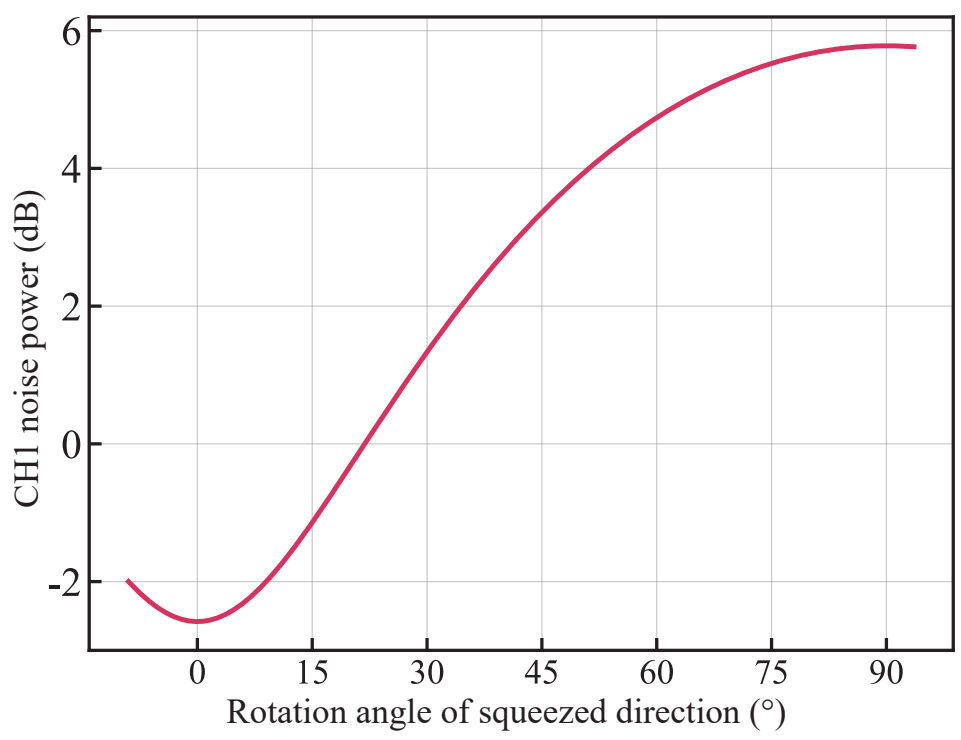

Fig. S6 Calculated noise power of the quadrature for a combined beam of two identical squeezed states (with squeezing level of 2.58 dB and anti-squeezing level of 5.76 dB). The squeezing angle of one squeezed state is fixed, while the squeezing angle of the other is varied.

Squeezing angle

In the main text, we described the experiments of measuring the squeezing of two combined (at the cell output) channels with distinct squeezing angles. Theoretically, we calculate the total quantum noise for two squeezed states with different phases in the squeezed vacuum. The results are presented in Fig. S6, which are in good agreement with Fig. 5(b) of the main text. The degree of squeezing reaches maximum when the relative phase of two squeezed vacuum state is 0°, and reaches minimum when the relative phase is 90°. When the squeezing direction of the two states are aligned, the total variance $\mathrm{Var}(X) = \mathrm{Var}(X_1) = \mathrm{Var}(X_2)$. In contrast, when the squeezing directions are orthogonal (separated by $n\pi/2$), the variance $\mathrm{Var}(X) = (\mathrm{Var}(X_1) + \mathrm{Var}(P_2))/2$.

Supplementary Note 3: Additional experimental results

Balanced homodyne detection and shot-noise calibration

Quantum quadrature noise is measured with a balanced homodyne detector (Thorlabs PDB450A; transimpedance gain, 10^5 V/A), using the x-polarized pump field as the local oscillator (LO). Each squeezing measurement is preceded by an independent shot-noise calibration at the corresponding LO power. A polarizing beam splitter (PBS) separated the y-polarized squeezed vacuum field (reflected port) from the x-polarized LO (transmitted port). During shot-noise calibration, coherent vacuum field enters the second PBS input port, and the recorded noise is taken as the shot-noise reference. By varying the relative phase between the squeezed vacuum field and the LO, the quantum noise of arbitrary quadratures is measured, while ensuring that the LO power is equal to that used for the corresponding shot-noise calibration. The noise power spectral density is recorded as a function of LO power from 0.5 to 12 mW with fixed spectrum-analyser settings. To quantify the shot-noise scaling, the noise power

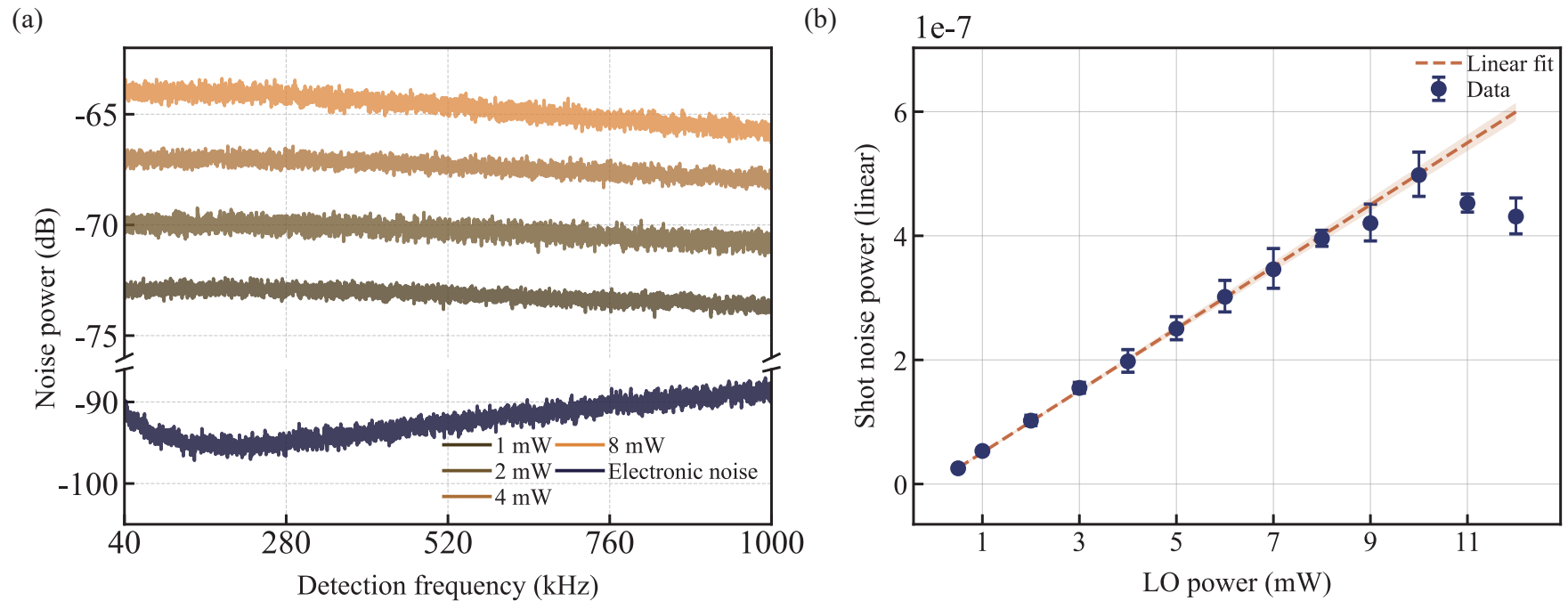

Fig. S7 Noise characterization of balanced homodyne detection. (a) Representative raw noise spectra measured with the balanced homodyne detector, including the electronic noise floor and shot-noise spectra at several LO powers. (b) Shot-noise power versus LO power. The data points are obtained by averaging the spectrum around the analysis frequency 180 kHz, and the dashed line is a linear fit in linear-power units. Error bars indicate the standard deviation within the averaging window, and the shaded region around the dashed line shows the fit uncertainty.

is extracted by averaging the spectrum over a narrow band centred at 180 kHz, chosen as a representative analysis frequency. A linear fit of the noise power (in linear units) versus LO power confirms shot-noise-limited operation. The electronic noise floor remains significantly below the shot-noise over the full data acquisition bandwidth, indicating a negligible contribution to the reported squeezing data. For an LO power of 3.188 mW, the detector imbalance is 0.31%. Representative raw noise spectra at several LO powers, together with the electronic noise floor, are shown in S7(a), and the corresponding noise variance versus LO power, the linear fit and its uncertainty are shown in S7(b).

Influence of the laser beam size

In addition to the parameters discussed in Fig. 2 of the main text, laser beam size is another critical factor determining the squeezing performance of the array. As shown in Fig. S8(a), the maximum achievable squeezing varies with the beam size, with an optimal value of about 0.5 mm for the 30-channel array. This is due to a trade off between competing effects. A larger beam size strengthens the inter-channel coupling but reduces the optical power density for a fixed total power. Meanwhile, a smaller beam increases the power density but eventually leads to saturation, which degrades the squeezing.

The interplay between the beam size and the laser power is further detailed in Fig. S8(b), which plots the noise power of a chosen channel against the array's beam size for two different input powers (3 mW and 4 mW per channel) in a 30-channel array. For beam sizes below 0.8 mm, a lower power of 3 mW yields superior squeezing. In this regime, the higher total power at 4 mW induces more spontaneous emission noise that degrades the squeezing. Conversely, for beams larger than 1.2 mm, the higher power of 4 mW becomes advantageous. Here, the system is limited by the efficiency of the four-wave-mixing process. The higher 4 mW power is required to achieve sufficient nonlinear interaction because of the lower optical density at larger beam sizes. This

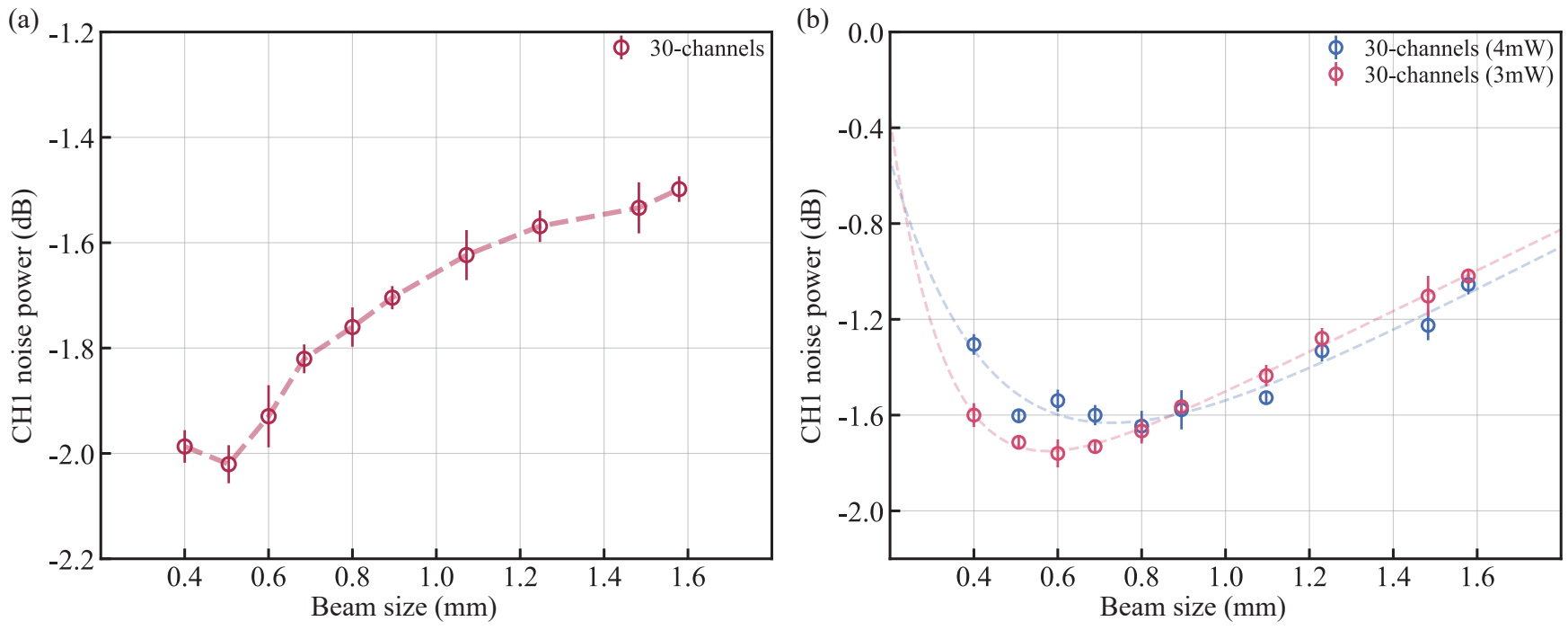

Fig. S8 Experimental light squeezing in a 30-channel array versus the laser beam size. (a) The optimal squeezing for various beam sizes, 0.400 mm, 0.500 mm, 0.600 mm, 0.689 mm, 0.800 mm, 0.895 mm, 1.097 mm, 1.230 mm, 1.483 mm, and 1.579 mm, with the corresponding optimal laser power of 1.75 mW, 2 mW, 2.25 mW, 2.5 mW, 2.75 mW, 3.2 mW, 3.5 mW, 3.5 mW, 4.0 mW, and 3.5 mW per channel, respectively. The experiment is conducted at a cell temperature of 63.7°C. (b) The squeezing versus beam size for fixed input powers of 3 mW and 4 mW (per channel) respectively, using the same set of beam sizes as in (a). The detection frequency is 160 kHz, and error bars represent the standard deviation from more than six repeated measurements. The noise power is normalized to the shot noise level (0 dB).

identifies the overall optimal squeezing performance for the array corresponds to the regime of relatively lower total power and smaller beam size.

Investigation of the anti-squeezed quadrature

In Fig. 4 of the main text, we show the squeezed and anti-squeezed quadrature noises versus the array's channel numbers, at relatively lower laser power (1 mW per channel). Here Fig. S9 extends these results to higher input light power (3 mW per channel). At 3 mW, the noise power in the squeezed quadrature decreases first and then rises as the number of channels in the array increases, while the noise power in the anti-squeezed quadrature consistently decreases. The numerical calculations are in good qualitative agreement with the experimental trends.

Optical phase distribution of the array

In the main text, we mentioned that the relative phase between the x-polarized field component and the squeezed vacuum determines the squeezing angle of the ellipse on the Poincaré sphere for the polarization squeezed state. Therefore, if the squeezing angles of all channels in the array are the same, and if the input x-polarized (pump) array are in phase, then we could infer that the squeezed vacuum array are in phase too. In order to prove that the pump array are in phase, we take images of both the near-field and far-field planes by a CCD camera. The far-field plane, representing the Fourier transform of the near-field, can reveal the information of the phase distribution of the array. When the optical channels are in phase, interference in the far field produces a well-defined intensity pattern [1]; conversely, random phases result in a disordered distribution. Due to the finite size of the lens and the relatively large spacing between the optical channels in the array, spherical aberration affects the imaging

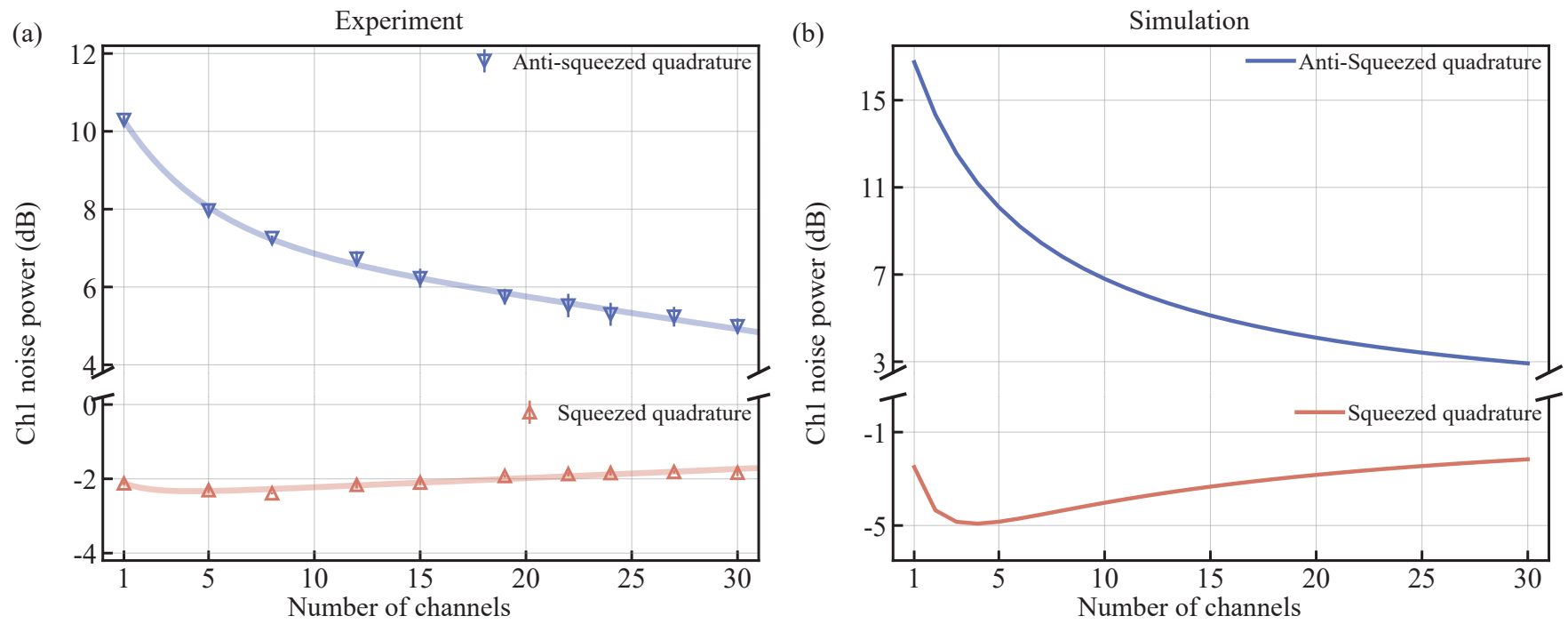

Fig. S9 Experimental and numerical noise power of the squeezed and anti-squeezed quadratures versus the array's channel numbers, for laser power of 3 mW per channel. The beam size is 0.505 mm, and the cell temperature is 63.7°C. The detection frequency is 180 kHz, and the noise power is relative to the shot noise level (0 dB). Error bars are obtained from more than six repeated measurements. The numerical results are obtained under the same parameters as specified above, employing a five-level model with a Rabi frequency of 76.6 MHz.

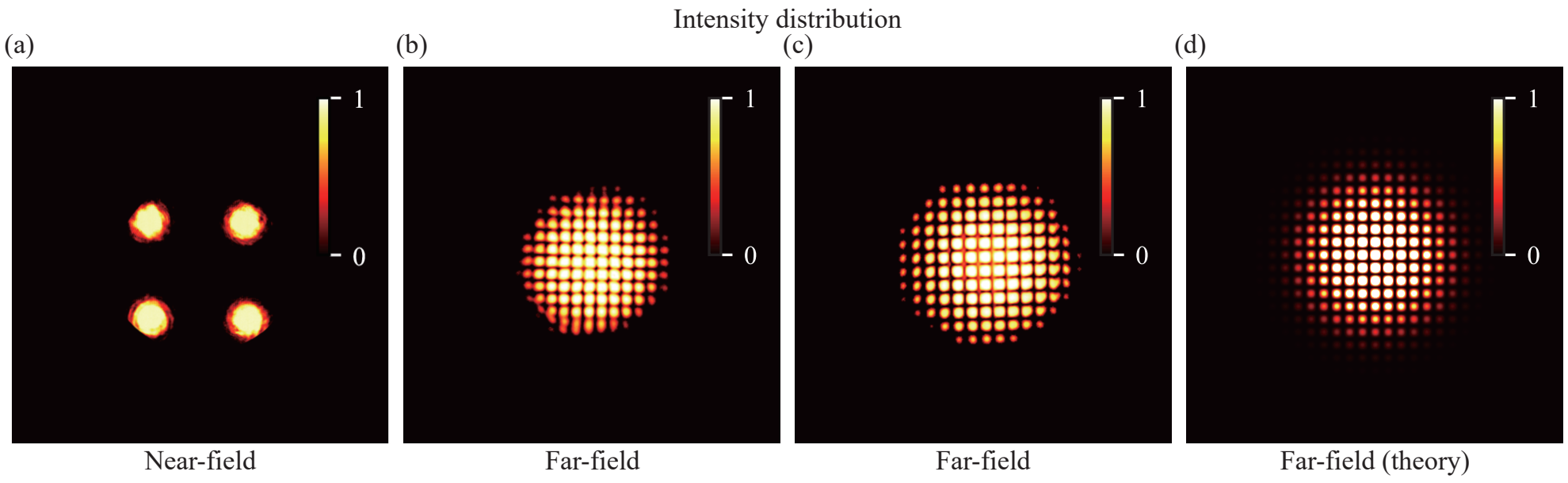

Fig. S10 Near-field and far-field images of 4-beam lattice in the 30-channel array. (a) Measured near-field intensity distribution of four adjacent beams in the array, showing a square lattice configuration with uniform beam size and intensity. Each channel is represented by a dot captured by a camera. (b) Measured far-field intensity distribution of a 4-beam lattice before entering the rubidium vapor cell. (c) Measured far-field intensity distribution of a 4-beam lattice after exiting the rubidium vapor cell. (d) Theoretically calculated far-field intensity distribution of a in-phase 4-beam lattice.

quality. Therefore, we randomly select (by an aperture) four adjacent optical channels arranged in a 2×2 square lattice out of the 30-channel array for measurement. The near-field intensity distribution is shown in Fig. S10(a), indicating uniform power across all channels. While the far-field intensity distribution (before the beams enter the rubidium vapor cell) is shown in Fig. S10(b), demonstrating that the optical array maintains near-perfect identical phases. After propagation through the rubidium vapor cell, we again arbitrarily select four channels for imaging, with the results presented in Fig. S10(c), confirming that the array retains its phase stability. Fig. S10(d) shows the theoretically calculated far-field intensity distribution of a 4-beam lattice where all the light beams have the same phase. It resembles the experimentally measured far-field intensity distribution pattern, indicating that the pump array are in phase.

Supplementary Note 4: Routes to improved performance of the squeezed array

In this section we point out, mainly through numerical simulations, the main factors affecting the squeezing level for the array and routes for future improvements. Overall, the guiding principle for larger squeezing in the array is to increase the total number of atoms, by increasing the atomic density (higher vapor cell temperature), using a longer or wider vapor cell. Then under a particular set of conditions of cell geometry and temperature, the laser power and detuning should be optimized accordingly.

Temperature dependence and wall coating

The maximal achievable squeezing level depends on the strength of the nonlinear atom-light interaction, which connects to the atomic vapor density and hence the cell temperature. Increasing the temperature enhances the optical depth (OD) and can improve squeezing. This dependence is evident in the experimental data in the inset of Fig. 2(b): raising the temperature from 50°C to 63.7°C yields ∼1 dB increase in squeezing, with the optimal pump laser power per beam changing from 0.75 mW to 2 mW. We emphasize that the temperature (63.7°C) optimally chosen in our experiment is limited by the paraffin coating on the cell's inner wall, and can be further increased if the coating material could tolerate higher temperatures. When the temperature approaches the melting point of the coating (typically 60–70°C for the paraffin in our cell), its capability of preserving the atomic coherence during wall collisions degrades [2], leading to higher spin relaxation rates. In addition, temperature-dependent modifications of atom-surface interactions, including adsorption and desorption dynamics, can reduce the effective vapor density [3]. Consequently, these effects impose a practical upper limit for the operating temperature of the squeezed array.

We numerically calculate how the array squeezing can improve with increasing temperature, assuming that the coating property does not degrade, and the temperature is not too high to cause decoherence due to atom-atom collisions [4]. As shown in Fig. S11, our model reproduces the experimentally observed trend of squeezing improvement with higher temperature (below 63.7°C). The simulation results suggest that squeezing can continue to improve between 63.7°C and 75°C. We note that, for larger OD, the onset of saturation shifts to higher pump laser powers, which results in a larger optimal operating laser power. For example, our simulation shows that as the temperature increases from 50°C to 75°C, the optimal power changes from ∼0.75 mW to ∼2.75 mW, while the corresponding squeezing level becomes higher.

This temperature-dependent saturation behavior occurs simply because more photons (higher total laser power in the cell) are needed to saturate more atoms (higher cell temperatures). It also accounts for the experimentally observed trends of array squeezing versus the number of channels shown in Fig. 2(b). At 55°C the squeezing degrades monotonically with increasing number of channels (under fixed per-channel power), indicating early onset of saturation. Whereas at 63.7°C the squeezing initially improves with the number of channels before reaching a plateau, suggesting that more atoms for the high temperature case delays the saturation.

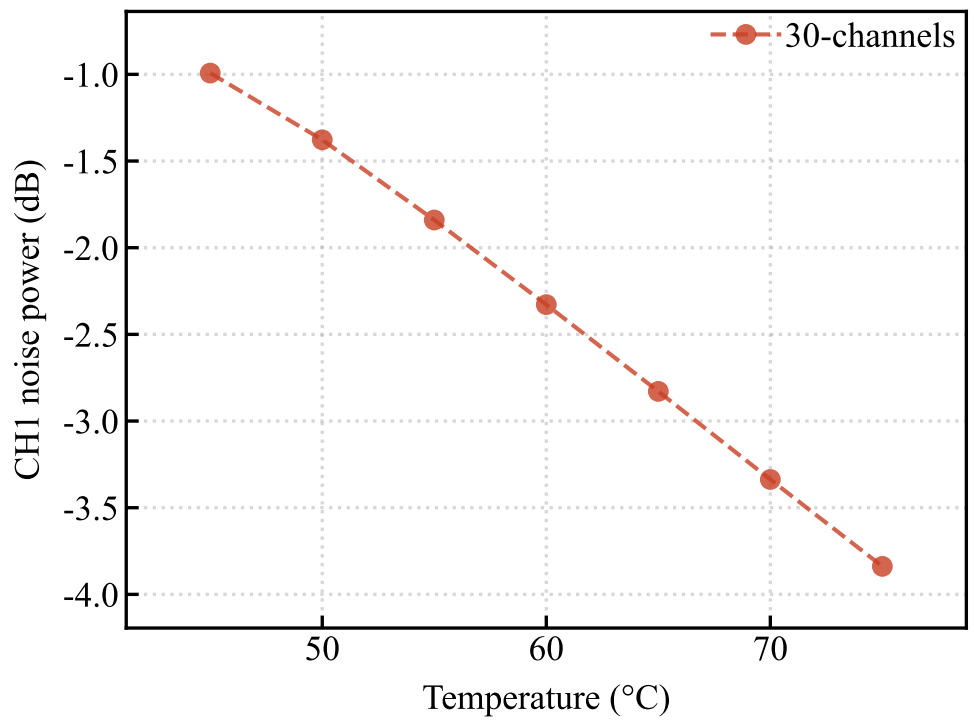

Fig. S11 Numerical results for the squeezed quadrature noise power of CH1 in a 30-channel array as a function of temperature where each channel in the array has the same input power. The laser frequency is resonant with $|5S_{1/2}F = 2\rangle \rightarrow |5P_{1/2}F' = 1\rangle$. The detection frequency is fixed at 160 kHz, and the noise power is normalized to the photon shot noise level (0 dB level). The cell geometry is fixed (length: 7.5 cm, diameter: 2.5 cm). For each temperature, the optical power per beam is chosen close to its corresponding optimal value to produce the best achievable squeezing under that condition. The powers used are 0.75 mW, 0.75 mW, 1 mW, 1.25 mW, 1.75 mW, 2.25 mW, and 2.75 mW for temperatures of 45°C, 50°C, 55°C, 60°C, 65°C, 70°C, and 75°C, respectively. The results show that higher temperature leads to higher optimal squeezing while the corresponding optimal laser power is higher, consistent with delayed onset of saturation for more atoms.

In addition, we note that previous PSR squeezing experiments in Rb vapor cell (without wall coating) were conducted in the 70–85°C range [5], which qualitatively agree with our numerical results favoring higher temperatures. Accessing this higher temperature regime in our platform requires replacing the paraffin coating with a thermally more stable alternative such as octadecyltrichlorosilane (OTS) that can withstand temperatures up to ∼170°C [6]. However, for PSR that involves near-resonant interaction and ground state coherence, a too-high temperature will cease to benefit squeezing because absorption and spin-exchange induced decoherence are severe [4].

Effect of the vapor cell length

Extending the vapor cell length increases the OD and strengthens the nonlinear atom-light interaction. Meanwhile, due to more atoms in a longer cell, more photons (higher total laser power in the cell) are required to saturate the atoms. For instance, as shown in Figure. S12, at 63.7°C, our numerical simulation indicates that increasing the cell length from 7.5 cm to 12 cm (with cell diameter fixed at 2.5 cm) improves the maximum squeezing from ∼2.7 dB (at 1.5 mW/channel) to ∼3.95 dB (at 3 mW/channel). We note that there is a limit for the cell length because excess noise due to spontaneous emission can build up during light propagation along the cell and eventually harm the squeezing. Therefore, the optimal cell length also depends on the laser power, cell temperature etc.

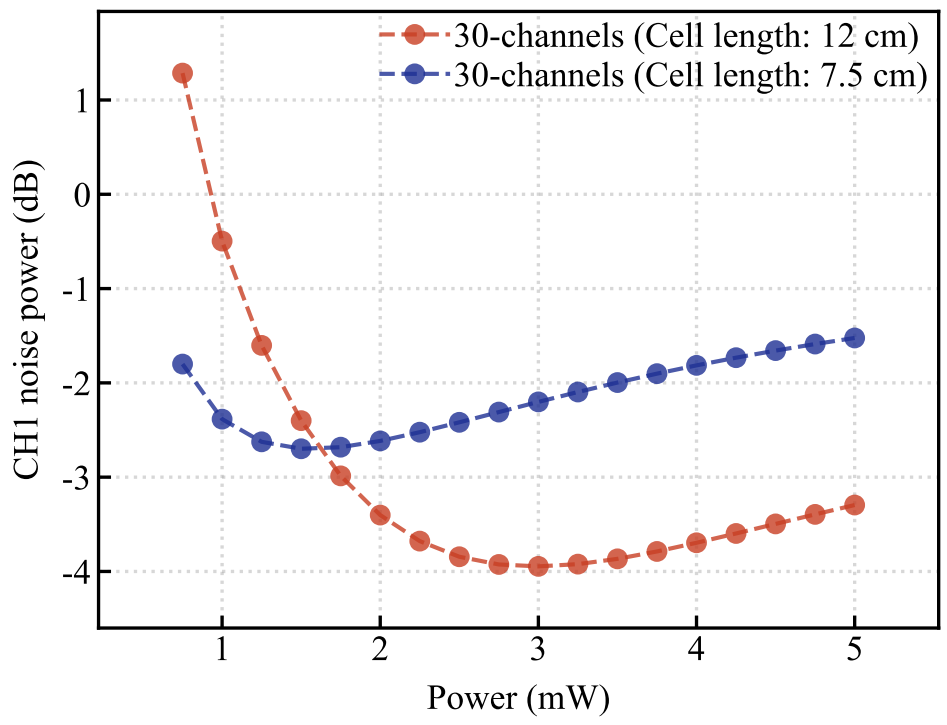

Fig. S12 Squeezing at different cell lengths. Numerical results for the squeezed quadrature noise power of CH1 in a 30-channel array as a function of optical power per channel, for cell lengths of 7.5 cm (blue) and 12 cm (red). The laser is resonant with $|5S_{1/2}F = 2\rangle \rightarrow |5P_{1/2}F' = 1\rangle$. The detection frequency is fixed at 160 kHz, and the noise power is normalized to the photon shot noise level (0 dB level). The cell diameter is fixed at 2.5 cm, and temperature is 63.7°C. The results show that increasing the cell length shifts the optimal power to higher values and enables a higher maximum squeezing.

Influence of the width of the vapor cell

In paraffin-coated vapor cells, atomic diffusion constantly redistributes ground-state population and coherence across the entire cell volume, enabling all atoms in the cell to collectively participate in the nonlinear atom-light interaction. As atoms in the optical channels are pumped into the trapped states (other hyperfine levels) and cease to participate the PSR process, fresh atoms can diffuse into the channels from the surrounding unilluminated region, which help to sustain the nonlinear optical process. Consequently, the saturation threshold is affected by the total atom number in the cell rather than the local atoms within any individual channel.

For a fixed array size, increasing the cell width or the diameter creates a larger bath for fresh atoms which can better replenish atoms within the optical channels. This would raise the saturation threshold in terms of the per-channel laser power. Figure. S13 presents the squeezing versus laser power for two different cell diameters, showing that both the optimal power and peak squeezing increase with the cell diameter: at 63.7°C, increasing the cell diameter from 2.5 cm to 4 cm (cell length fixed at 7.5 cm) can improve the maximal squeezing from ∼2.7 dB (reached at 1.5 mW/channel) to ∼3.87 dB (reached at 2.5 mW/channel). This confirms that the total atom number in the cell influences the onset point of the saturation behavior. Finally, we note that while wider cell is preferred for the squeezed light array, in practice, a wider vapor cell requires more stringent control of the residual magnetic field inhomogeneity within the shielding.

Multi-parameter optimization

The squeezing performance reported in the main text was observed in the coated vapor cell available in the lab, and does not represent the ultimate limit of our scheme. As described above, the cell temperature and cell geometry such as the length and width,

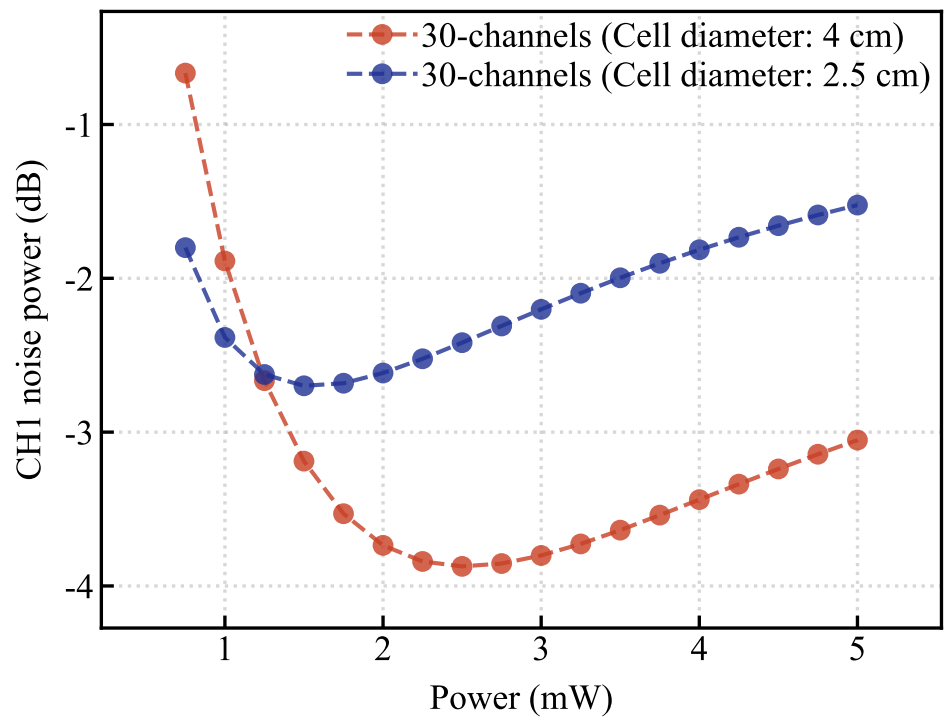

Fig. S13 The influence of cell width on array squeezing. Numerical results for the squeezed quadrature noise power of CH1 in a 30-channel array as a function of optical power per channel, for cell diameters of 2.5 cm (blue) and 4 cm (red). The laser is resonant with $|5S_{1/2}, F{=}2\rangle \rightarrow |5P_{1/2}, F'{=}1\rangle$. Detection frequency: 160 kHz; noise power normalized to the photon shot noise level (0 dB). Cell length: 7.5 cm; temperature: 63.7°C. Increasing cell diameter raises the total atom number, shifts the optimal pump power to higher values, and yields larger peak squeezing.

provide room for improvement. In addition, laser parameters such as the laser power and laser detuning should be optimized accordingly because the saturation threshold depends on the total atom number. Overall, it is a problem of multi-parameter optimization.

The PSR process used here is a coherence-enhanced nonlinear optical process where the ground state coherence is established by EIT, a resonant atom-light interaction. However, there is still some room for laser detuning optimization, with a minor effect on the squeezing level compared to the zero detuning case. When on resonance, the atomic medium provides the strongest nonlinear response, and our experimental optimization confirms that squeezing is maximized near zero laser detuning under our primary operating conditions (63.7°C, 2 mW/channel). At higher temperature and especially higher laser power, although the nonlinearity is higher, optical pumping also becomes significant, then shifting the laser slightly away from resonance can mitigate resonant absorption while still maintaining an efficient nonlinear interaction for squeezing. As shown in Fig. S14, the calculated optimal detuning is different for two laser powers of 1 mW/channel and 2 mW/channel, for 70°C and 55°C. However, the squeezing at optimal red-detuning is only slightly different than at zero-detuning. We note that, since the optimal laser power itself varies with the total atom number through the cell temperature and cell geometry as described above, the optimal detuning also depends on these parameters.

Another parameter affecting the obtained squeezing is the detection frequency, given a finite bandwidth for light squeezing in general. In our scheme, due to the motion of the atoms it is mainly the array's total laser power averaged across the cell cross-section that determines the squeezing bandwidth. Therefore, theoretically, there is higher squeezing at lower detection frequency, and when we make quantitative comparisons of squeezing under various conditions, the detection frequency is specified. Since the optimal laser power depends on the total number of atoms via the cell

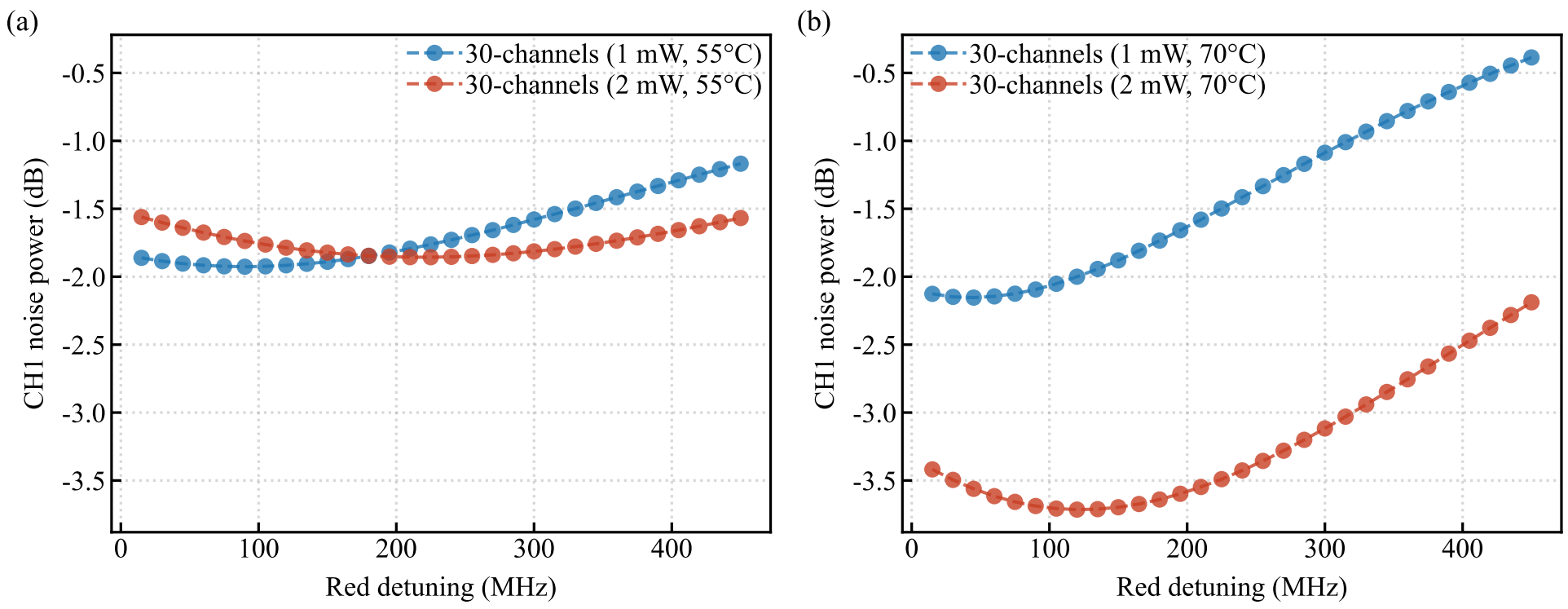

Fig. S14 Effect of laser detuning on light squeezing. Numerically calculated squeezed-quadrature noise power for CH1 in a 30-channel array as a function of the value of the red detuning of the laser, for per-channel optical powers of 1 mW (blue) and 2 mW (red) at two temperatures. Zero detuning corresponds to resonance with the $|5S_{1/2}, F{=}2\rangle \rightarrow |5P_{1/2}, F'{=}1\rangle$ transition. Detection frequency: 160 kHz; Cell length: 7.5 cm; Cell diameter: 2.5 cm; Noise power is normalized to the photon shot noise level (0 dB). (a) Temperature: 55°C. (b) Temperature: 70°C. It can be seen that the optimal laser detuning has a dependence on laser power and temperature.

geometry and temperature, the squeezing spectrum varies correspondingly with these conditions. Figure. S15 shows calculated squeezing spectra at various optical powers for 63.7°C, illustrating the change of squeezing bandwidth with the total laser power. In the present work, experimentally, the detection frequency was chosen near 180 kHz due to dominate technical noises at lower frequencies such as magnetic field noises coupled to the atoms. Importantly, when the cell becomes wider, due to motional averaging of the atoms, the average laser intensity decreases, leading to narrowed squeezing bandwidth. In this case, squeezing detected at lower frequency can be significantly higher than that at higher frequency, which is why the detection frequency is chosen to be 60 kHz in Fig. S16 when showcasing improved squeezing.

Building upon the above analysis of how squeezing depends on various parameters, we next showcase the simulation results of significantly improved array squeezing under certain sets of parameters of operating temperature, cell geometry (including cell length and width), laser power, laser detuning, and detection frequency. As shown in Fig. S16, at 70°C, by increasing the size of the vapor cell, in particular by using a longer cell and a larger cell cross section, the achievable squeezing of the 30-channel array can exceed 8 dB. We note that, the results in Fig. S16 are obtained at a fixed per-channel power for all cell sizes in order to isolate the effect of cell geometry, even though that power may not be optimal operating power for all cell sizes. In a larger cell, the atom number participating in the nonlinear interaction is higher, raising the saturation threshold to higher laser power. Consequently, a condition that is near-optimal for a larger cell may correspond to a significantly sub-saturated regime for a smaller cell, and vice versa. Therefore, jointly optimizing the cell temperature (including the coating material), cell geometry and laser parameters represent an

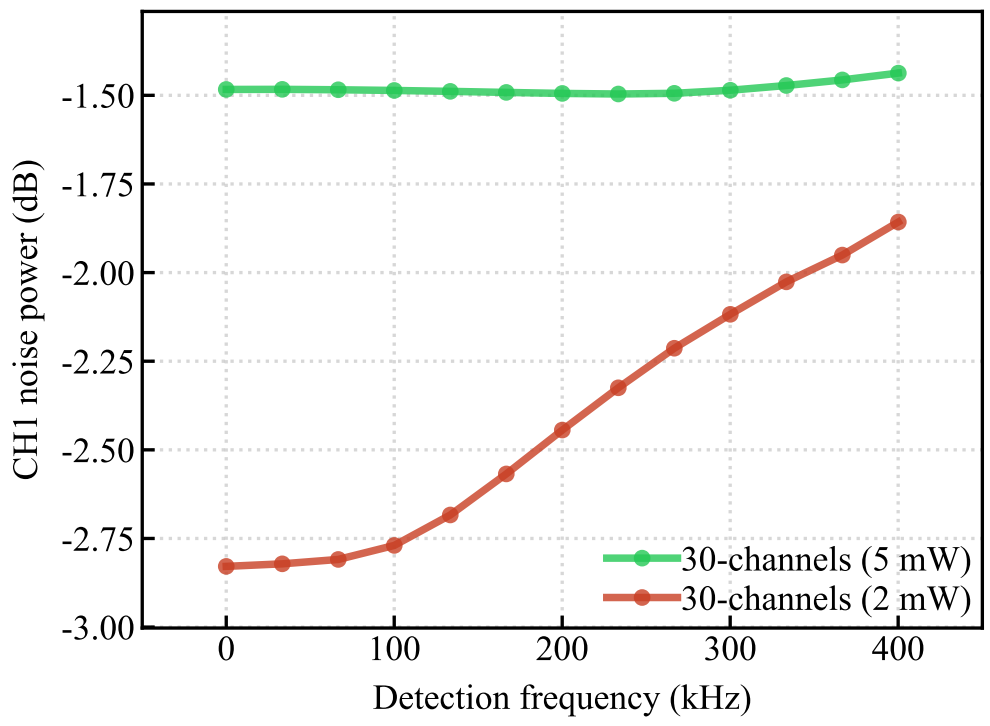

Fig. S15 Noise spectrum for different laser power in the array. Numerically calculated squeezed-quadrature noise spectrum for CH1 in a 30-channel array, shown for per-channel optical powers of 2 mW (red) and 5 mW (green). The laser is on resonance with the $|5S_{1/2}, F{=}2\rangle \rightarrow |5P_{1/2}, F'{=}1\rangle$ transition. Noise power is normalized to the photon shot noise level (0 dB). Cell length: 7.5 cm; diameter: 2.5 cm; temperature: 63.7°C. It can be seen that there is higher squeezing at lower detection frequency.

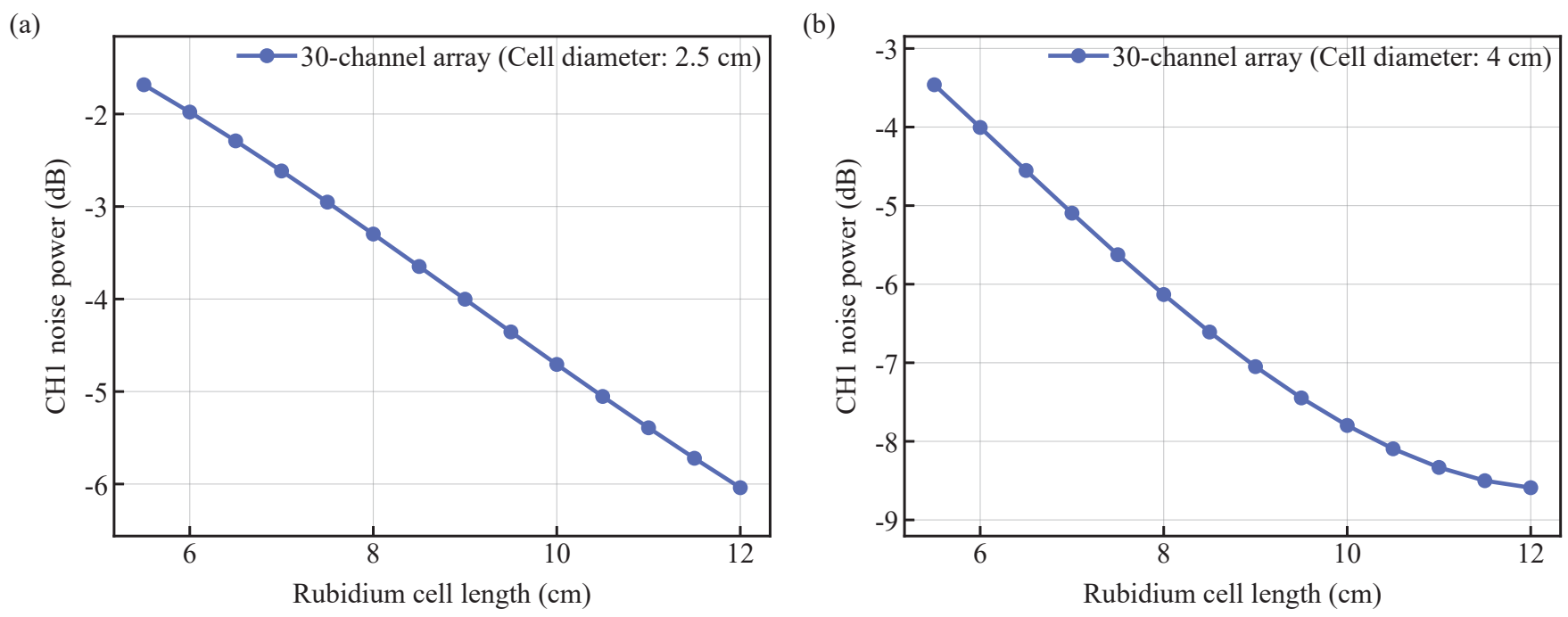

Fig. S16 Significantly improved array squeezing results for higher temperature and larger vapor cell. Numerical simulation for the squeezed quadrature noise power of CH1 in a 30-channel array as a function of the length of vapor cell at temperatures of 70°C, where each channel in the array has the same input power of 5 mW (Rabi frequency of 98.9 MHz). The laser frequency is red-detuned by 120 MHz from $|5S_{1/2}F = 2\rangle \rightarrow |5P_{1/2}F' = 1\rangle$. The detection frequency is fixed at 60 kHz, and the noise power is normalized to the photon shot noise level (0 dB level). (a) Cell diameter: 2.5 cm. (b) Cell diameter: 4 cm.

effective and practical route to substantially enhancing array squeezing beyond the levels demonstrated in our current experiment.

References

[1] Mahler, S., Goh, M. L., Tradonsky, C., Friesem, A. A. & Davidson, N. Improved phase locking of laser arrays with nonlinear coupling. *Phys. Rev. Lett.* **124**, 133901 (2020).

[2] Li, W. *et al.* Characterization of high-temperature performance of cesium vapor cells with anti-relaxation coating. *J. Appl. Phys.* **121**, 063104 (2017).

[3] Atutov, S. N., Benimetskiy, F. A., Plekhanov, A. I., Sorokin, V. A. & Yakovlev, A. V. Diffusion of rb atoms in paraffin-coated resonant vapor cells. *Eur. Phys. J. D* **71**, 12 (2017).
[4] Happer, W. Optical pumping. *Rev. Mod. Phys.* **44**, 169–249 (1972).
[5] Zhang, M. *et al.* Multipass configuration for improved squeezed vacuum generation in hot rb vapor. *Phys. Rev. A* **96**, 013835 (2017).
[6] Seltzer, S. J. & Romalis, M. V. High-temperature alkali vapor cells with anti-relaxation surface coatings. *J. Appl. Phys.* **106**, 114905 (2009).
